# Microstructures amplify carotenoid plumage signals in tanagers

**DOI:** 10.1038/s41598-021-88106-w

**Published:** 2021-04-21

**Authors:** Dakota E. McCoy, Allison J. Shultz, Charles Vidoudez, Emma van der Heide, Jacqueline E. Dall, Sunia A. Trauger, David Haig

**Affiliations:** 1 Department of Organismic and Evolutionary Biology, Harvard University, 26 Oxford Street, Cambridge, MA 02138 USA; 2 Informatics Group, Harvard University, 38 Oxford Street, Cambridge, MA 02138 USA; 3 Museum of Comparative Zoology, Harvard University, 26 Oxford Street, Cambridge, MA 02138 USA; 4 Ornithology Department, Natural History Museum of Los Angeles County, 900 Exposition Blvd, Los Angeles, CA 90007 USA; 5 Harvard Center for Mass Spectrometry, Harvard University, 52 Oxford Street (B2), Cambridge, MA 02138 USA

**Keywords:** Sexual selection, Evolution, Optics and photonics, Optical materials and structures, Biomaterials

## Abstract

Brilliantly-colored birds are a model system for research into evolution and sexual selection. Red, orange, and yellow carotenoid-colored plumages have been considered honest signals of condition; however, sex differences in feather pigments and microstructures are not well understood. Here, we show that microstructures, rather than carotenoid pigments, seem to be a major driver of male–female color differences in the social, sexually-dimorphic tanager genus *Ramphocelus*. We comprehensively quantified feather (i) color (using spectrophotometry), (ii) pigments (using liquid chromatography–mass spectrometry (LC–MS)), and (iii) microstructures (using scanning electron microscopy (SEM) and finite-difference time-domain (FDTD) optical modeling). Males have significantly more saturated color patches than females. However, our exploratory analysis of pigments suggested that males and females have concordant carotenoid pigment profiles across all species (MCMCglmm model, female:male ratio = 0.95). Male, but not female, feathers have elaborate microstructures which amplify color appearance. Oblong, expanded feather barbs in males enhance color saturation (for the same amount of pigment) by increasing the transmission of optical power through the feather. Dihedral barbules (vertically-angled, strap-shaped barbules) in males reduce total reflectance to generate “super black” and “velvet red” plumage. Melanin in females explains some, but not all, of the male–female plumage differences. Our results suggest that a widely cited index of honesty, carotenoid pigments, cannot fully explain male appearance. We propose that males are selected to evolve amplifiers—in this case, microstructures that enhance appearance—that are not necessarily themselves linked to quality.

## Introduction

Why are so many birds colorful? To investigate this evolutionary “why,” we study both the physical mechanisms of color (pigments and structures) and the evolutionary mechanisms which favor colorful signals over time (selective forces). If we can fully understand the physical basis of traits, we may gain insights into their evolutionary history.

Three overlapping selective pressures shape the evolution of colorful mating displays. First, coloration may facilitate species identification, essential to avoid sterile hybrids and wasted mating efforts^1^. Second, colorful ornaments may reflect aesthetic preferences in the choosing sex^2^, perhaps shaped by a Fisherian runaway process^3^ or by selection on another domain such as foraging—termed “sensory bias”^4^. Third, and most commonly researched, color may indicate individual quality (“honest signaling theory”), either as (i) an index of health^5–9^ or (ii) as a costly signal (e.g., due to parasite load^10^, general handicap^11^, or social costs^12–18^).

Honest signaling theory is the most prominent explanation for colorful bird plumages, and red, orange, and yellow carotenoid-colored birds are a “textbook example of an honest signal”^6^. Carotenoids must be eaten by vertebrates rather than synthesized and may be scarce in nature. Carotenoids are correlated with some (but not all) individual quality measures^5,6,19^, and may be an index of proper metabolic function rather than a costly signal^20^ (see research on finches *Taeniopygia guttata*^7^, crossbills *Loxia curvirostra*^8^, and house finches *Haemorhous mexicanus*^21^).

Carotenoid-colored birds are used widely to research sexual selection and honest signaling, but the full physical basis of color in males compared to females is not yet understood (i.e., pigmentary and structural differences). It is useful to note that nanostructures and pigments are generally well understood in bird coloration^22^, but *micro*structures are less well studied. Microstructures are barb or barbule features greater than one micrometer (µm) but less than one millimeter (mm) in size^23–31^; in contrast, nanostructures^32–35^ such as ordered melanosomes are less than 1 µm in size (ranging from tens to hundreds of nanometers (nm)). See diagram in Fig. 8 for a schematic overview of how pigments, nanostructures, and microstructures contribute to feather color. Several studies show that microstructures are important in bird color; microstructures (i) make carotenoid-based colors glossy or matte^26^, (ii) create green mirrors in the African Emerald Cuckoo *Chrysococcyx cupreus*^25^, (iii) enhance melanin to generate super black appearance in 15 families of birds^28,29^, (iv) differ between male and female fairy wrens *Malurus spp.*^24^, (v) cause angle-dependent colors due to boomerang-shaped barbs in bird-of-paradise *Parotia lawesii*^31^, (vi) impact whether reflectance is directional of diffuse^30^, (vii) cause gloss in cassowaries *Casuarius casuarius* due to a thick rachis^23^, (viii) vary alongside black and brown melanin-based color in Corvidae^27^, and more. Outside of birds, many organisms use microstructures to enhance the impact of pigments: flowers use conical epidermal cells to make richer petal colors^36–39^ and microstructures contribute to super black color in peacock spiders^40^, stick insects^41^, and snakes^42^. To what extent may microstructures, rather than pigments, explain color differences between carotenoid-colored male and female birds? The answer may help us determine which selective forces favor colorful ornaments.

**Figure 1:**
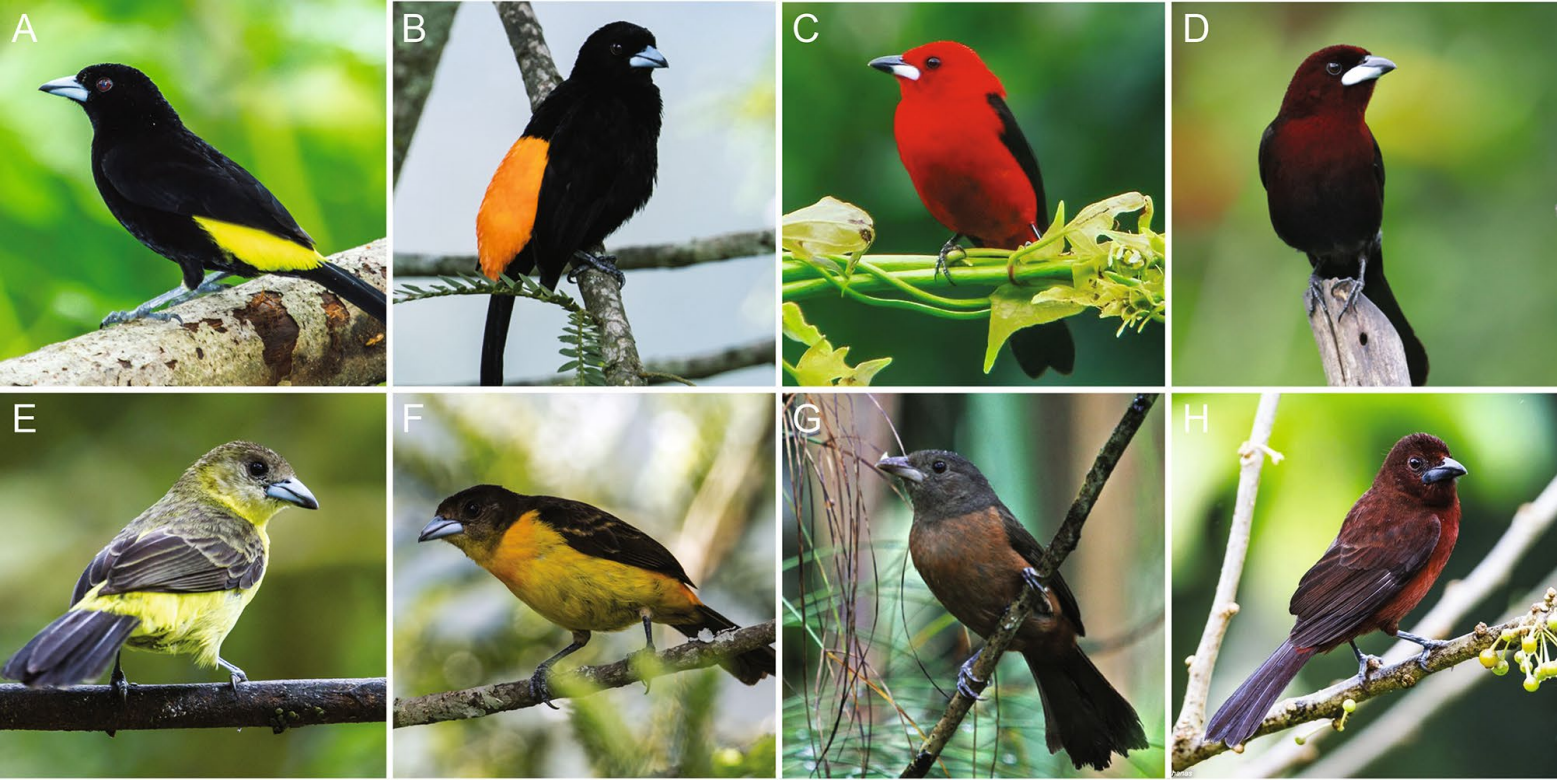
Male *Ramphocelus* tanagers (top row) have more striking carotenoid-based coloration than females (bottom row). (**A**) *R. flammigerus icteronotus* male with vivid yellow and deep black plumage. (**B**) *R. flammigerus icteronotus* female. (**C**) *R. flammigerus* male with vivid orange and deep black plumage. (**D**) *R. flammigerus* female. (**E**) *R. bresilius* male with bright red plumage. (**F**) *R. bresilius* female. (**G**) *R. carbo* male with velvet red and deep black plumage. (**H**) *R. carbo* female. All photos are credit Nick Athanas—www.antpitta.com.

Here, we explore the physical basis of carotenoid color in male versus female birds, and thus draw inferences about the evolutionary selective pressures which favor colorful signals. We focus on the social, sexually dimorphic *Ramphocelus* tanagers, a useful clade for questions of visual signaling^43,44^ and mating behavior^45–47^. These tanagers have carotenoid-based coloration ranging from bright yellow to dark matte red in males, while females are relatively duller (Fig. 1). Using scanning electron microscopy (SEM), finite-difference time-domain (FDTD) optical modeling, pigment extraction, liquid chromatography–mass spectrometry (LC–MS), spectrophotometry, and digital light microscopy, we document the physical and chemical basis of feather color in both males and females. From these exploratory analyses, we make inferences about the dynamics of mate choice over evolutionary time. We find preliminary evidence that microstructures enhance carotenoid-based signals in males.

## Material and methods

### Specimens

*Ramphocelus* tanager specimens were selected from the Ornithology Collection at the Harvard Museum of Comparative Zoology (MCZ; specimen numbers are listed in Table S1). We selected N = 20 total intact male and female specimens, one from each species in the genus *Ramphocelus* plus one subspecies with visually divergent plumage that had full species status in the past^48^: Crimson-collared tanager, *Ramphocelus sanguinolentus;* Masked crimson tanager, *Ramphocelus nigrogularis;* Crimson-backed tanager, *Ramphocelus dimidiatus;* Huallaga tanager, *Ramphocelus melanogaster;* Silver-beaked tanager, *Ramphocelus carbo;* Brazilian tanager, *Ramphocelus bresilius;* Passerini's tanager, *Ramphocelus passerinii;* Cherrie's tanager, *Ramphocelus costaricensis;* Flame-rumped tanager, *Ramphocelus flammigerus;* Lemon-rumped tanager, *Ramphocelus flammigerus icteronotus.* Taxonomy is according to the Clements Checklist v.2017^49^. We selected only one individual per species because we were primarily focused on interspecific variation, but we confirmed the repeatability of pigment extractions using 3 individuals per species for two species (see below). Colorfastness of carotenoids varies tremendously between bird species, with some species such as *Cardinalis* (Cardinalidae; closely related to the *Ramphocelus* tanagers) retaining their color in darkness “almost indefinitely”^50^. To ensure that collection methods, preparation, handling, and age of the specimen did not bias our results, we selected males and females within a species from the same collecting trips (specimen details in Table S1; information about specimens available from Harvard Museum of Comparative Zoology MCZBase). Age of the specimen had no discernible effect on carotenoid presence.

We designate *R. flammigerus, R. f. icteronotus*, *R. passerinii*, and *R. costaricensis* to be the “rumped” tanagers because they form a clade with vivid color restricted to the rump. We designate the clade of tanagers with color on their whole body to be the “whole body” clade (*Ramphocelus nigrogularis*, *Ramphocelus dimidiatus*, *Ramphocelus melanogaster*, *Ramphocelus carbo*, *Ramphocelus bresilius*). *R. sanguinolentis* is the sister to all others.

### Spectrophotometry

**Figure 2:**
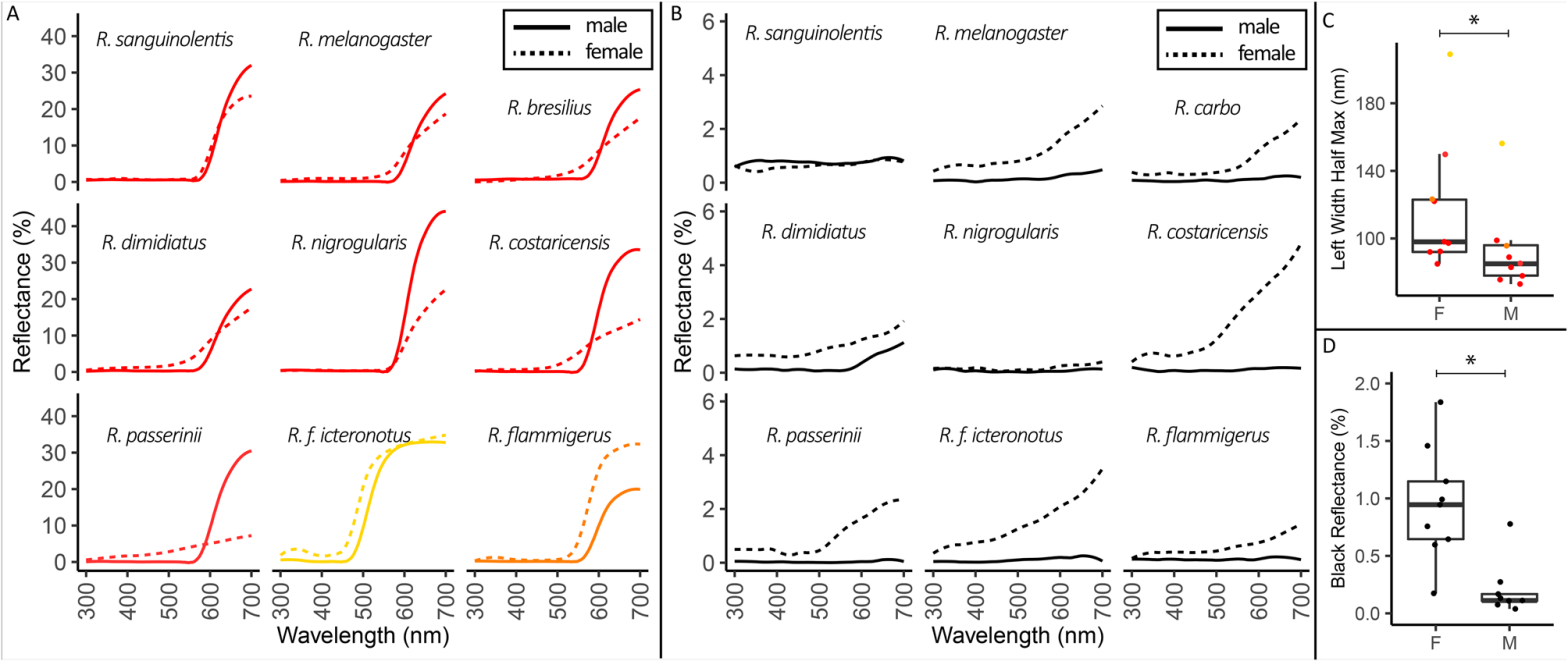
Male Ramphocelus tanagers have significantly more saturated colors and darker blacks than females. Spectrophotometry results for 90° incident light. (**A**) Males (solid lines) have brighter and more saturated colors for all species except *R. flammigerus* and *R. f. icteronotus*. We included the body region of each male and female with the max reflectance, which was rump for all species except male *R. sanguinolentis* (crown), male *R. bresilius* (crown), female *R. bresilius* (stomach), female *R. dimidiatus* (stomach), female *R. costaricensis* (breast), and female *R. passerini* (throat). We excluded *R. carbo* from this plot, because it is dark red rather than brilliant; N = 9. (**B**) Males (solid lines) have a darker black color, with a flatter reflectance curves, on their backs compared to females (dashed lines) for all species except *R. sanguinolentis*. We measured the black back of each species except *R. dimidiatus*, for which we measured the black throat. We excluded *R. bresilius*, which is all red except for its tail and wings; N = 9. (**C**) For a measure of saturation, leftwards width at half maximum reflectance, males had significantly more saturated color than females (phylogenetic two-sample paired t-test; p-value = 0.0072, 95% CI: [13.0, 38.1]). Narrower peaks (i.e., smaller value for left width at half maximum reflectance), indicate a more saturated color. (**D**) For a measure of darkness, total integrated reflectance under the curve, males had significantly darker black regions than females (phylogenetic two-sample paired t-test; p-value = 0.0066, 95% CI: [0.38, 1.11]). Complete spectrophotometry results are in Supplemental Data 1. Integration time was 100 ms, and each line in the spectra (**A**,**B**) and point in the boxplots (**C**,**D**) represents the average of three replicates within the same plumage patch.

We performed spectrophotometry using an OceanOptics PX2 with a pulsed xenon light source, and OceanOptics USB4000, and an OceanOptics Spectralon White Standard. We used an integration time of 100 ms, averaged 5 scans, and set a boxcar width of 5. We measured three replicates per plumage patch for each of N = 20 individuals (male and female from 10 species). The replicates were averaged for plotting and for statistical comparisons (Fig. 2). For each specimen, we measured the bird at 7 locations: crown, back, rump, dorsal tail, throat, breast, and belly. Using an OceanOptics reflectance probe holder, we measured reflectance for 90° incident light and for 45° incident light. These two angles of incidence allow us to determine the directionality and structural absorption potential of the plumage patches.

We wished to compare saturation of colorful regions and brightness of black regions between males and females. Brightness depends on total reflected light; therefore, we calculated brightness as the integral of the area under the reflectance curve between 300 and 700 nm divided by 400. Saturation depends on the steepness and narrowness of a color peak^51^; therefore, we calculated an index of saturation by modifying typical full width at half maximum calculations^52^. Specifically, we calculated the maximum reflectance, divided that value by two, and calculated the leftwards width of the reflectance curve between the maximum and half-maximum reflectance values. To compare darkness of black regions, we selected the body region of each male and female bird that was darkest (usually backs; see Fig. 2). To compare saturation of colorful regions, we selected the body region of each male and female bird that had the highest reflectance (usually rumps; see Fig. 2).

### Carotenoid Identification (Mass Spectrometry)

We prepared feathers for pigment extraction using a simple mechanical procedure. First, we washed all feathers in hexane and allowed them to air dry. Next, we trimmed off the entirety of colored portions of barbs and barbules from three feathers. We carefully weighed them, then placed these barbules with 0.3 mL methanol in a screw cap micro tube in the presence of 10 ceramic beads (2 mm). We subjected these tubes to bead beating in a FastPrep FP120 for three cycles, each for 45 s at level 6.0. We centrifuged the resulting mixture in an Eppendorf centrifuge 5417R for 5 min at 3000 RCF, then transferred the supernatant (ensuring that no carotenoid-based color remained in the tube) and dried it under a stream of nitrogen. All samples were kept at -80 °C until analysis. The samples were resuspended in 100 µL of acetonitrile:methanol (1:1) and transferred to micro inserts in amber glass vials; immediately after resuspension, the samples were analyzed.

Initially, we took 9 feathers per species (three feathers from each of three specimens for two species) to ensure that we had enough material; for all additional species we took 3 feathers from a single patch on a single individual, which proved sufficient. In order to ensure that results were repeatable between individuals of a single species, we extracted pigments from two additional individuals for each of two species—*R. flammigerus* and *R. flammigerus icteronotus*. The pigment profiles were significantly correlated (Fig. S3, Linear regression output for *R. flammigerus*: slope = 0.88, SE = 0.066, R^2^ = 0.87, p < 0.0005. Linear regression output for *R. f. icteronotus*: slope = 0.96, SE = 0.054, R^2^ = 0.92, p < 0.0005), indicating that our extraction and characterization procedure was repeatable across individuals of a species. See Statistics section for details. Excluding the repeated measures of *R. flammigerus* and *R. flammigerus icteronotus*, we extracted pigments from N = 32 plumage regions in males and females, from 3 feathers per plumage region. To select which feathers to use for carotenoid extractions, we plotted all reflectance spectra for the following regions of each bird—crown, back, rump, tail, throat, breast, stomach—and observed that rump was nearly always the most saturated region (complete data in Supplemental Data). To avoid confounding our dataset of structures and pigments with many different body regions, which may have differently shaped feathers, we thus selected rump feathers for pigment extraction from every bird where the rump was a saturated color (versus a brown or a black). For the dark-colored species *R. melanogaster* and *R. carbo,* the rump was not a saturated color- therefore we selected the colorful patches of these birds, which were: male *R. melanogaster* throat and upper throat; female *R. melanogaster* throat and chest; male *R. carbo* breast; female *R. carbo* crown. In addition, we wanted to investigate variation in bright, specular red versus matte, dark red within single birds. We already had this contrast from *R. melanogaster* and thus also sampled rump and throat from *R. dimidiatus* male and female. Finally, we were curious about the females with both orange and yellow coloration, so in addition to rump, we sampled throat from *R. costaricensis* and breast from *R. flammigerus.*

We acquired pigment standards for 8-apo carotenal, Canthaxanthin, Astaxanthin (Sigma Aldrich), Lycopene (Santa Cruz Biotechnology), and Alloxanthin (ChromaDex). Carotenoid contents were analyzed using an Ultimate 3000 LC coupled with a Q-Exactive Plus hybrid quadrupole-orbitrap mass spectrometer (Thermo Fisher Scientific). The LC was fitted with a Kinetex C18 (2.6 µm, 100 Å, 150 mm × 2.1 mm, Phenomenex) column and the mobile phases used were MA: Acetontrile:Methanol 85:15 and MB: 2-propanol. The gradient elution started with 4 min of 5% MB, then to 100% MB in 11 min, followed by 4 min at 100% MB, 1 min to get back to 5% MA and 4 min re-equilibration at 5% MB. The flow was kept constant at 0.185 mL min^−1^, and 10 µL of samples were injected. Electrospray ionization was used in positive ion mode. The mass spectrometer was operated at 70,000 resolving power, scanning the m/z 150–1500 range, with data-dependent (top 5) MS/MS scan at 17,500 resolution, 1 m/z isolation and fragmentation at stepped normalized collision energy 15, 35, and 55. The data were first manually analyzed to identify peaks of ions with accurate masses corresponding to potential carotenoids within a 5 ppm mass accuracy threshold. The potential carotenoids included both known molecules, and those with a similar molecular formula based on their accurate mass and mass defect. MS/MS fragmentation spectra were then compared with the standards available or to MS/MS database entries within METLIN (-metlin.scripps.edu) to assign identity when standards were available or putative identity based on fragmentation pattern similarity. Lastly, peaks corresponding to carotenoids or carotenoids-candidates were integrated using Quan Browser (Xcalibur, Thermo Fisher Scientific). Pigments were grouped into “families”, based on their accurate mass and relation to standards and fragmentation similarities.

LCMS allowed us to precisely characterize which molecules comprised the plumage carotenoids across all species in the genus *Ramphocelus*, but it is important to note that this is an exploratory analysis. LCMS is useful to explore trends in sex differences across species, but the signal strengths of different carotenoid molecules cannot be directly compared. As one measure to control for this, we incorporated pigment family as a random effect in our statistical models (see below). Also, we focused on free carotenoids rather than considering carotenoid esters (see^53^; see Limitations).

We assessed feathers for melanin presence using digital light microscopy and, following^54^, by analyzing parameters of the reflectance curves (Supplementary Methods, Equation S1).

### SEM & feather microstructure

In preparation for SEM, we mounted a single feather from each specimen (N = 20 feathers) on a pin using a carbon adhesive tab. We sputter-coated the feathers with ~ 12 nm of Pt/Pd at a current of 40 mA. We performed scanning electron microscopy on an FESEM Ultra55.

Using ImageJ, we made multiple measurements on each SEM image, including maximum barb width, top-down barb width (barb width when viewing the feather from directly above), barbule width, inter-barbule distance, barb-barbule angle, and barbule length. We also coded feathers according to whether the barbules were strap shaped or cylindrical, and whether the barbules emerged from the plane of the feather (i.e., had “3-D” structure).

### Optical modelling: finite-difference time-domain simulations of (i) angled barbules and (ii) oblong, expanded barb

To approximate the optical effect of the observed microstructures, we performed finite-difference time-domain (FDTD) simulations using the commercially available software Lumerical FDTD. FDTD is a versatile method for simulating the interaction of light with objects by computationally solving Maxwell’s equations. FDTD simulations calculate the spatio-temporal electromagnetic field distribution that results when an initial pulse of light is launched onto, and interacts with, simulation objects within a bounded measurement domain—the “Yee cell” method^55^.

For each simulation, our general setup was as follows. A plane wave of light (ranging from 400 to 700 nm) was normally incident (y-direction) on an idealized feather cross-section in the x–y plane. We performed 2D simulations without incorporating pigment because (i) we wished to isolate the effect of structure in a simple, interpretable manner and (ii) the mathematical characterization of carotenoid absorption is not well understood when multiple pigments are mixed in a feather, as is the case here (but future work focusing on absorption spectra could use the Kramers–Kronig relationship to characterize the refractive indices of each pigment^56^). The simulation domain was bounded at the horizontal edges of the y plane by perfectly matched layers (which are artificial absorbing layers) and at the vertical edges by periodic boundary conditions (which repeat the unit of our single-feather simulation at both edges to simulate a feather plumage composed of many side-by-side feathers). Frequency domain field monitors were placed above and below the structure to collect the reflected and transmitted light, respectively. We used a mesh size of 0.025 × 0.025 µm^2^.

We conducted two sets of simulations for two apparently important microstructural features, as determined by the PCA described below: (i) angled barbules (hypothesized to decrease reflectance) and (ii) oblong and expanded barb (hypothesized to increase saturation).

(i) Angled Barbules. We hypothesized that angled barbules caused lower total reflectance. Therefore, for our optical models of angled barbules, we assessed total reflectance based on structure alone without considering the contribution of melanin. That is, we calculated the quantitative decrease in feather reflectance from angled barbule structure alone. To focus only on the impact of surface reflections and light’s path through the feather after surface diffraction, we extended the bottom feather surface to beyond the bottom vertical perfectly-matched layer boundary in the y-plane. This was a measure to eliminate reflections from the underside of the feather because in life, feathers are arranged in complex stacks in a plumage, which we did not simulate; feathers are flush with other feathers (i.e., the bottom of a feather would be a feather-feather interface, not a feather-air interface). For these simulations of angled barbules, we simulated a feather with hemispherical barb (radius 12.5 µm) and barbules 80 µm long varying in angle from 0 to 80°. We present the average and standard deviation reflectance for 15 barbule angles (15 simulations).

(ii) Oblong, Expanded Barb. We hypothesized that more oblong, expanded barbs cause more saturated colors. We could not measure color saturation directly, because carotenoid absorption is not well characterized for the molecules of interest. Therefore, for this exploratory analysis we measured a proxy of color saturation: optical power transmission through the barb, a measure of how much light energy passes through an area. The greater the total optical power transmission through pigment (i.e., light passing through the pigment), the greater the light-pigment interactions. For example, when flowers use conical cells to focus light onto pigment, increasing the optical power transmission therethrough, the resulting color is more saturated^37^. The barb is carotenoid-pigmented in *Ramphocelus* tanagers (Fig. S5B-G) and other carotenoid-colored birds^50^, often with carotenoids scattered throughout the keratin substrate^57^. Therefore by calculating increase in power transmission through the barb, we can approximate the structural enhancement of pigmentary color due to barb size and shape in males. For our simulations of the male-typical barb versus female-typical barb, we simulated an average female feather barb based on our measurements: 22.2 µm tall by 18.9 µm wide—and an average male feather barb: 39.8 µm by 27.7 µm.

For the oblong, expanded barb simulations, we implemented four strategies to test the robustness of our results. First, we measured optical power transmission through an entire feather, including the feather bottom (feather-air interface). Second, because in life feathers are packed into a plumage with no bottom feather-air interface, we simulated a feather truncated at its base, excluding structural effects of the feather bottom. Third, in life feathers often contain vacuoles, so we simulated male and female feathers with a 10 µm-diameter circular air vacuole in the center. Fourth, for all simulations we calculated the total optical power transmission through four 7.5 × 7.5 µm^2^ square regions of the barb: (i) center, (ii) side, offset 15 µm in the X direction, (iii) 45° angle, offset 10 µm in both X and Y directions, and (iv) top, offset 20 µm in the Y direction. We performed a one-way paired t-test, using the R function t.test (alternative = “less”), of our 11 total power transmission measurements to check whether males do indeed have higher power transmission than females (the total of 11 measurements comes from four measurements (barb center, side, top, 45° angle) for each of three simulations, minus the barb center for the vacuole simulation). We present total optical power transmission for all 11 measurements of male and female feathers, as well as results of the t-test, in Table S6, and in Fig. 6 we plot power transmission for 700 nm light (results were similar for other wavelengths of light) from one simulation (entire feather, no vacuole).

### Phylogeny

**Figure 3:**
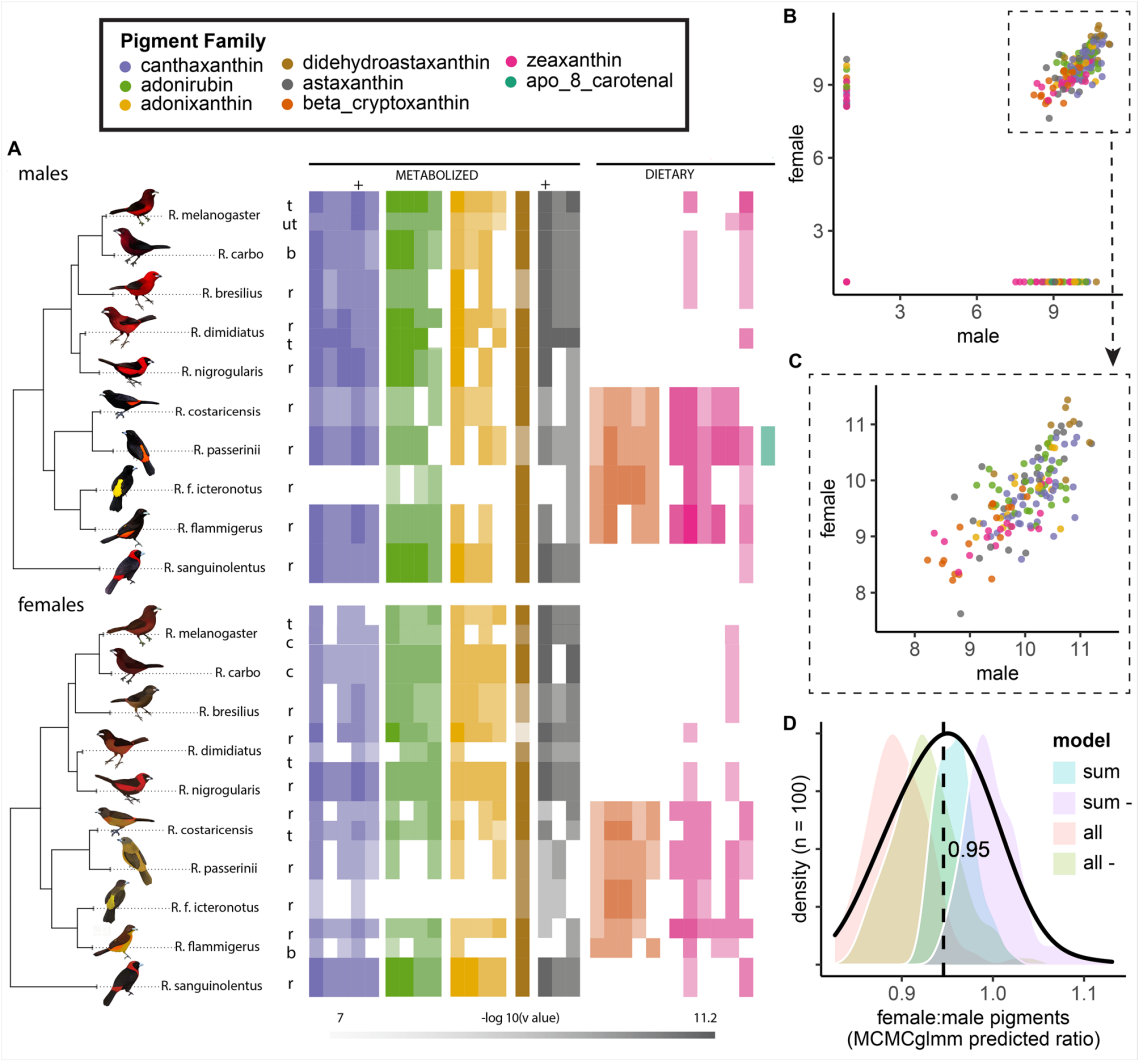
Across species, males and females have significantly correlated carotenoid profiles with a mean female:male ratio of 0.95. (**A**) Relative log-transformed presence of pigments across birds for males (N_species_ = 10, N_patches_ = 12) and females (N_species_ = 10, N_patches_ = 14). Color saturation indicates normalized signal strength of a given pigment molecule within a bird, where pale is least and rich is most. Relative intensity comparison can therefore only be made within each molecule (column). Some comparisons are possible within each family, because response factors of isomers are expected to be similar. Because standards were not available for all molecules, some molecules were identified based on accurate mass, retention time, MS/MS spectra, and the literature (see Table S4). (**B,C**) Male versus female carotenoid pigments; each point represents the signal strength (a proxy for presence and amount) of a given pigment in both a male and female of one species. All values are log-transformed and normalized. Male and female pigment profiles are significantly correlated (MCMCglmm model including pigment family and phylogeny as random effects; for fixed effect male pigment as a predictor of female pigment, posterior mean = 0.55, 95% CI = [0.4434, 0.67], N_effective_ = 1800, p < 0.001, DIC = 1578.2; when pigments were summed by family (Fig. S4, posterior mean = 0.74, 95% CI = [0.52, 0.96], N_effective_ = 1800, p < 0.001, DIC = 377.9 ). C. Zoom in on male versus female carotenoid pigments as shown in B but, for visualization, restricted to points where male and female values were both greater-than-0. (**D**) MCMCglmm model predictions for the ratio between male and female pigment levels; mean ratio was 0.95. Models included were all (all isomers), all- (all isomers minus apo-8-carotenal), sum (summed isomers within pigment families), and sum- (summed isomers within pigment families minus apo-8-carotenal). Abbreviations: t: throat; ut: upper throat; r: rump; b: breast; c: chest. Artwork in male silhouettes credit Gabriel Ugueto; female silhouettes are modified by Allison Shultz from original art by Gabriel Ugueto.

Several recent studies have examined the phylogeny of *Ramphocelus* tanagers^58–60^. Unfortunately Burns and Racicot^59^ and Burns et al.^58^ did not include *R. f. icteronotus*, and Hackett^60^ did not include *R. melanogaster* or *R. dimidiatus*, but the two studies combined had *cytochrome b* (cyt b) sequences available for all species. To construct a phylogeny, we downloaded cyt b sequences from NCBI for all *Ramphocelus* species, and six outgroups from the Tachyphoninae clade (*Tachyphonus coronatus, T. rufus, T. phoenicius, Eucometis penicillate, Lanio fulvus, and T. luctuosus*). NCBI accession numbers are available in Table S2. We aligned the sequences with MAFFT v. 7.313^61^, and constructed the best-scoring maximum likelihood phylogeny with Raxml v. 8.2.10 using the GTRCAT model with 100 bootstrap replicates^62,63^. The resulting phylogeny confirmed the relationships found by previous studies, and so we extracted the monophyletic *Ramphocelus* clade for use in downstream analyses (Fig. 3).

### Statistics

To compare saturation and feather brightness in males and females, we performed phylogenetically controlled two-sample t-tests in R, using phyl.pairedttest from package phytools v. 0.6–44^64,65^. For these tests each of the three replicates per species and sex were averaged (Fig. 2).

To assess the correlation between male and female carotenoid pigment profiles across species while controlling for the influence of phylogeny, we fit generalized linear mixed models using the MCMCglmm package in R^66^. Such models can include phylogenetic effects and use Markov chain Monte Carlo sampling^67–69^. Phylogenetic effects and pigment family were included as random variables. It was necessary to include pigment family, because our carotenoid quantification method—LCMS—measures signal intensity, not amount, and different types of carotenoids cannot be directly compared. For the results reported herein, we specified the prior [list(R = list(V = 1, nu = 0.002), G = list(G1 = list(V = 1, nu = 0.002), G1 = list(V = 1, nu = 0.002)))]; we also ran models with medium and high belief priors to ensure that model outcomes were insensitive to prior parameterization. We used 1,000,000 iterations, a burn-in period of 100,000, and a sampling interval of 500. We assessed graphical diagnoses of model performance (plotting model_name$Sol and model_name$VCV), ensured that levels of autocorrelation were below 0.1 using autocorr(model_name$VCV), ensured that each model generated effective sample sizes of ~ 1000, and assessed phylogenetic signal by calculating lambda^67–69^. Additionally, we ran two sensitivity models: (i) excluding the pigment apo-8-carotenal (found only in male *R. passerinii*), and (ii) without the random effect of pigment family to check whether pigment family exerted a strong influence on model performance. Finally, because the isomers observed herein may not be relevant to phenotype, we ran all models again after summing all pigment isomer signals within a pigment family. To assess whether model outcome was sensitive to these decisions, we compared posterior means and Deviance Information Criteria, or DICs. As a further sensitivity test, and to investigate each pigment separately, we performed a phylogenetic paired t-test to check for differences between male and female pigment presence for each pigment family using the function phyl.pairedttest in package phytools^65^.

To derive a quantitative estimate of the relationship between male and female carotenoid pigment profiles, we used the function predict in the MCMCglmm package in R^66^, randomly subsampling our dataframe of male pigment values to predict female pigment values 100 times. During each sub-sampling iteration, we summed the model-predicted female values and divided them by the real male values to generate an estimate of female:male pigment ratio. We subsampled 50 data points for two all-isomer models and 20 data points for two summed-isomer models, and repeated each procedure 100 times, therefore producing 400 total estimates.

We performed principal component analyses (PCAs) on microstructure in males and females, using both standard and phylogenetic PCA methods in the software program R, version 3.4.3. We log transformed and centered all data. For standard PCAs we used function prcomp in R, both centering and scaling the data on feather microstructure (Table S3). PC1 and PC2 were extracted and used for visualizations; PC1 was used to assess correlations between males and females.

For phylogenetic PCAs of feather microstructure, we used function phyl.pca in the R package phytools^65^, with a lambda method of correlation, and PCA mode “cov” (covariance).

To assess whether male feather microstructure was correlated with plumage saturation, we performed a phylogenetic generalized least squares model (PGLS;^70,71^) using function gls from package nlme^72^. Specifically, we tested whether saturation correlated with PC2, which captured barbule width and oblong-ness.

We tested for correlations between the proportions of carotenoids detected (carotenoid signal, as peak area) in two replicates each of two species, *R. flammigerus* and *R. f. icteronotus*. First, for each individual, we normalized the signal for each carotenoid to account for variation in overall amounts of carotenoid detected (versus proportion) by dividing the signal of each carotenoid by the sample weight and then by the maximum signal detected for that individual. We then tested for a correlation between the two individuals of each species separately with a linear model. We performed the same procedure to test for correlations between the upper and lower throat of male *R. melanogaster* and the throat and rump of male *R. dimidiatus*.

We also tested for correlations between male and female feather structure using the PCA scores, which captured a large proportion of the variance. For microstructure, we tested PC1 and PC2, and used phylogenetic generalized least squares (PGLS;^70,71^) with a Brownian motion model to account for phylogeny. We could not use PGLS for multiple patches when measured, so we randomly selected one patch for each species. Analyses using the alternative patch were qualitatively similar, but not presented here. Results are robust to alternative phylogeny transformations (Brownian motion model, Ornstein–Uhlenbeck model). Note that these PC scores were taken from the non-phylogenetic microstructure PCA, and thus are directly comparable between males and females (both of which were included in the PCA).

Finally, we reconstructed the evolutionary history of carotenoid evolution for each pigment family for males and females. Because there might have been slight variations in isoform for different species, we decided to focus on carotenoid families as the most biologically meaningful variables. First, for each pigment family, we summed all isoforms for males and females, and log-transformed them (base 10). We removed any species that were missing data for both males and female for that family. We did the reconstruction with the contMap function in phytools v. 0.6–44^64,65^, which uses maximum likelihood to estimate states at internal nodes and interpolate these states along internal branches^73^.

## Results

### Male Ramphocelus tanagers have significantly more saturated colors and darker blacks than females

Spectrophotometry revealed a wide range of yellows, oranges, and reds in males and females, adjacent to blacks (Fig. 2, complete results in Supplementary Data). Vivid, highly saturated color patches in males typically reflected almost no light for short wavelengths before sloping sharply upwards to reflect long wavelengths in the yellow-orange-red space (Fig. 2A). In contrast, females of most species had colorful patches with a relatively more gradual upward slope, a greater-than-0 reflectance over a broad range of wavelengths, and a relatively lower peak reflectance (Fig. 2A). Males from multiple species had extremely low-reflectance black plumage, with broadband flat reflectance below 0.5%, in areas adjacent to bright color patches (Fig. 2B). Females had typical black plumage reflectances, with a slight increase in reflectance at higher wavelengths (Fig. 2B).

Males had significantly more saturated color than females (phylogenetic two-sample paired t-test; p-value = 0.0072, 95% CI: [13.0, 38.1]) based on a measure of saturation, leftwards width at half maximum reflectance (Fig. 2C). That is, they had narrower peaks. Males had significantly darker black regions than females (phylogenetic two-sample paired t-test; p-value = 0.0066, 95% CI: [0.38, 1.11]), where darkness was measured as total integrated reflectance under the curve (Fig. 2D).

Many males also had unusual “velvet red” coloration, which we define as plumages red in hue that reflect < 5% of incident light and are matte (absent specular reflections, similarly to super black plumages; Supplementary Data) in a manner similar to the super black plumages in other species (Fig. 2B). One species, *R. carbo*, has velvet red color on its whole body.

In general, many of the colored feathers were strong directional reflectors, such that reflected light differed in quantity (percent total reflectance) from 90° incident light to 45° incident light (Supplementary Data). For example, the velvet red patches on *R. carbo* increased their reflectance from ~ 0–1% to roughly 5–7% when the angle of light incidence changed from 90° to 45°. This characteristic, reflecting more light at a lower angle of incidence, is a hallmark of feathers with microstructures that impact absorbance and reflection^28^.

### Across species, males and females have significantly correlated carotenoid profiles with a mean female:male ratio of 0.95

Through these exploratory analyses, we identified 29 distinct molecules that matched the absorption spectra of carotenoids. These fell into 8 groups by monoisotopic molecular mass, with 1–6 different molecules in each pigment group, which we refer to as pigment families (Table S4). We mapped the relative abundances of all described molecules onto a tree for both males and females (Fig. 3A), performed ancestral state reconstructions for each pigment family (Fig. S1), and mapped all identified pigments onto a metabolic network that shows how different molecules can be modified within the body (Fig. S2).

Five of the molecules matched our purchased pigment standards and could be conclusively identified. For the remaining pigments, the molecule’s identity was inferred based on accurate mass, retention time, MS/MS spectra, and pigments commonly found in bird feathers as described in the literature. For example, it is difficult to distinguish between the two dietary carotenoids lutein and zeaxanthin. Using the metabolic networks presented in Morrison and Badyaev^74^ and LaFountain et al.^75^, we identify the molecules with the accurate mass 568.43 as zeaxanthin because none of the common avian derivates from lutein were found in any of our samples, while many derivates of zeaxanthin (and related metabolites) were found. If some or all of the detected molecules with this mass were lutein, it would not change the conclusions herein, because both are dietary pigments.

We found that dietary pigments zeaxanthin, β-cryptoxanthin, and apo-8-carotenal were found primarily in the rumped tanager clade (Fig. 3A, blue stars in Fig. S2) while the whole-body tanagers and *R. sanguinolentis* had primarily metabolized carotenoids. The “rumped” tanager clade had less metabolized pigments than the “whole-body” clade and *R. sanguinolentis* (Fig. 3A). This was affirmed through ancestral state reconstructions (Fig. S1). These mappings also showed qualitative concordance between male and female pigment profiles within a species, and qualitative concordance between pigment profiles of different regions within a bird (Fig. 3).

To assess the relationship between male and female carotenoid pigments, we conducted exploratory MCMCglmm analyses (but see Limitations). Female carotenoid pigment scores were correlated significantly with male for MCMCglmm models including all pigment isomers (Fig. 3A-C) and including summed pigment signals within a family (Fig. S4); (MCMCglmm model included pigment family and phylogeny as random effects; main model: for fixed effect male pigment as a predictor of female pigment, posterior mean = 0.55, 95% CI = [0.4434, 0.67], N_effective_ = 1800, p < 0.001, DIC = 1578.2; summed isomers model (Fig. S4): posterior mean = 0.74, 95% CI = [0.52, 0.96], N_effective_ = 1800, p < 0.001, DIC = 377.9). Models were insensitive to prior parameterization, autocorrelation was near < 0.1 for all^67^, and model results also did not change when we excluded pigment family as a random effect (suggesting pigment family is not exerting a strong influence). When we ran the model with summed pigment isomers, the DIC was significantly lower (DIC = 337.9 vs 1578.2), but those values cannot be directly quantitatively compared. For both all-isomer and summed-isomer models, the DIC improved significantly when we excluded apo-8-carotenal from the model, likely because this pigment was present only in male *R. passerinii.* Phylogenetic signal (calculated as lambda) was 0.039, suggesting a very low influence of phylogenetic structure on the data. When we predicted female:male pigment ratios using our top four models (Fig. 3D), we found a mean value of 0.95. This suggests that male and female pigment profiles are highly similar. The plots of the raw data (Fig. 3A-C; Fig. S4 for summed data plots) qualitatively support our quantitative conclusions based on MCMCglmm models: male and female tanagers have concordant pigment profiles. However, it is critical to note that we measured signal intensity—not pigment amount, directly—and therefore these results should be treated as exploratory findings across species (rather than precise findings within species).

As a simple sensitivity test, we performed a phylogenetic paired t test to investigate male versus female values for each pigment separately; we found no significant differences between males and females, supporting the results in the MCMCglmm.

### Some plumages incorporate melanin, particularly in females

In some cases, male and female birds differed perceptibly in not only saturation but also brightness and hue, which (unlike most changes in saturation) could be explained by melanin^76^. We found indirect evidence of melanin in regions from 8 females and 2 males (Fig. S5A; by analyzing reflectance curves) and direct microscopy evidence of melanin in five of six colorful females and one of six colorful males (Fig. S5B-G). Taken together, these exploratory results suggest that melanin pigments play a role in any unexplained differences in *brightness* or *hue* between male and female feathers.

### Males, but not females, have diverse and elaborate feather microstructure

**Figure 4:**
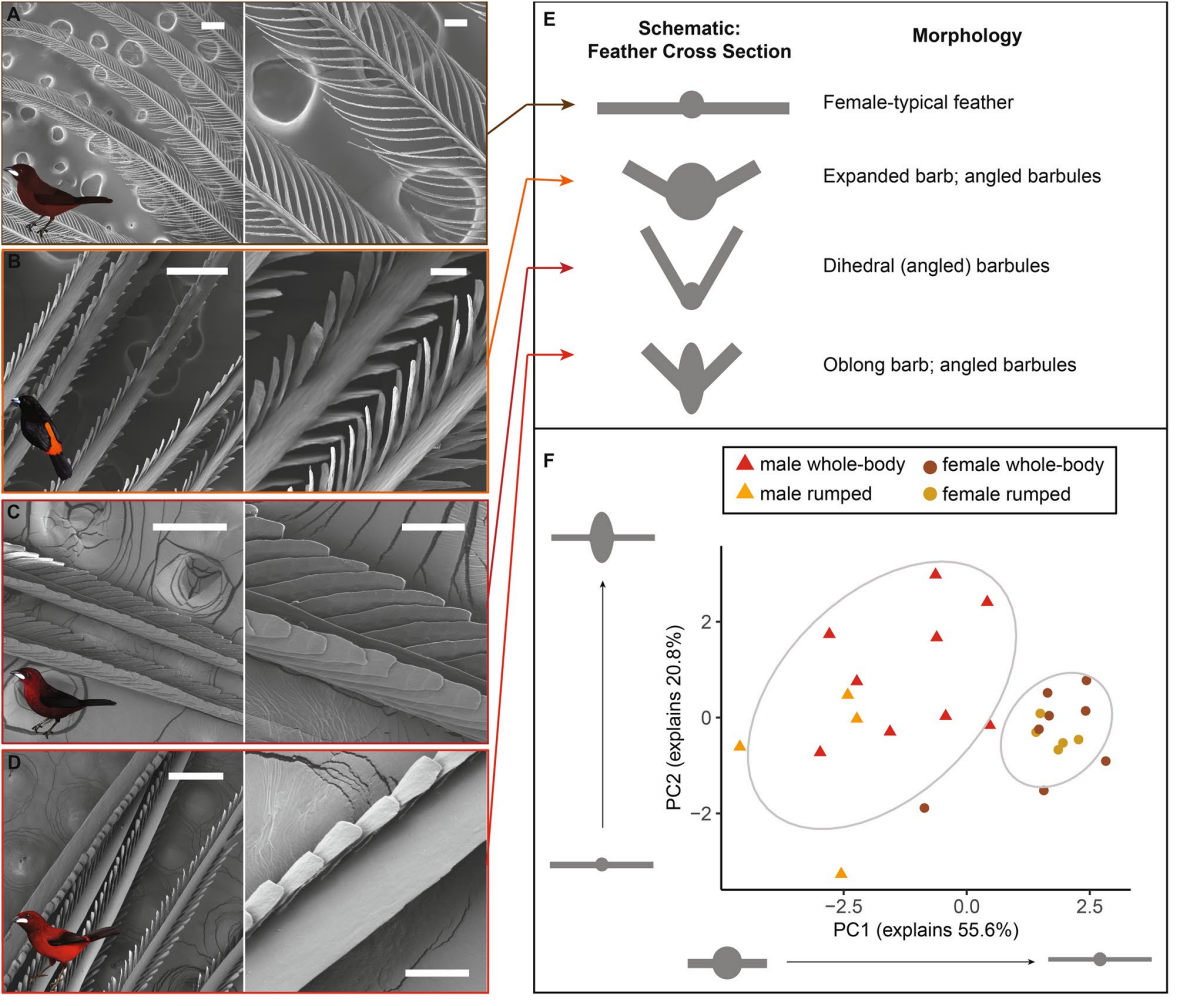
Males, but not females, have diverse and elaborate feather microstructure. (**A**) Female *R. carbo* red chest feather with typical simple morphology. (**B**) Male *R. passerinii* bright orange rump feather with expanded barb and strap-shaped barbules. (**C**) Male *R. carbo* velvet red back feather with dihedral barbules. (**D**) Male *R. dimidiatus* shiny red rump feather with expanded, oblong barb and strap-shaped barbules. Scale bars in left column are 200 µm; scale bars in right column (**B**,**D**,**F**,**H**) are 50 µm. Artwork in inset silhouettes credit Gabriel Ugueto; female silhouette in **A** is modified by Allison Shultz from original art by Gabriel Ugueto. E. Schematic illustrations of idealized cross-sections of each feather type in **A**-**D**. (**F**) PCA of log-transformed centered microstructural measurements for all patches from all males and all females, showing that males separate from females. Male and female microstructures are not correlated (PGLS with microstructure PC1 scores: coefficient = -0.023, SE = 0.17, p = 0.90). This PCA does not consider differences in three-dimensional structure, including dihedral barbule morphology; see Table 1.

Female *Ramphocelus* tanager feathers were mostly of standard microstructural appearance^29^. That is, feather microstructure usually looks the same as feather macrostructure, with simple cylindrical barbules extending from the central cylindrical barb, all in a single plane (Fig. 4A, Table 1, Table S3). The only female to exhibit broad variation was *R. sanguinolentis* (Table 1, Fig. S8, Table S3). Female *R. sanguinolentis* in fact had microstructural characteristics more typical of male feathers: angled, strap-shaped barbules, and an oblong expanded barb.

**Table 1:** Males, but not females, have diverse and elaborate feather microstructure. Male *Ramphocelus* tanagers have many atypical microstructural features compared to females. “3D barbules” refers to whether or not barbules extended upwards from the plane of the feather; “strap-shaped barbules” refers to whether barbules were flattened rather than classically cylindrical; “oblong barb” refers to whether barbs were taller than they were wide (numerical threshold: > median (max barb width)/(top-down barb width)); wide barb refers to whether the barb was expanded (numerical threshold: > median barb width value). Complete numerical measurements can be found in Table S3.

Species	Sex	Region	Angled barbules	Strap-shaped barbules	Oblong barb	Expanded barb
*R. bresilius*	F	Rump	–	–	–	Yes
*R. carbo*	F	Chest	–	–	Yes	–
*R. costaricensis*	F	Throat	–	–	–	–
*R. dimidatus*	F	Rump	–	–	–	–
*R. dimidatus*	F	Throat	–	–	Yes	–
*R. flammigerus*	F	Breast	–	–	–	–
*R. flammigerus*	F	Rump	–	Yes	–	–
*R. f. icteronotus*	F	Rump	–	Yes	–	–
*R. melanogaster*	F	Chest	–	–	–	–
*R. melanogaster*	F	Throat	–	Yes	–	–
*R. nigrogularis*	F	Rump	–	–	Yes	–
*R. passerinii*	F	Rump	–	–	Yes	–
*R. sanguinolentis*	F	Rump	Yes	Yes	Yes	Yes
*R. bresilius*	M	Rump	Yes	Yes	Yes	–
*R. carbo*	M	Back	Yes	Yes	Yes	Yes
*R. carbo*	M	Breast	Yes	Yes	Yes	Yes
*R. costaricensis*	M	Rump	Yes	Yes	–	Yes
*R. dimidatus*	M	Throat	Yes	Yes	–	Yes
*R. dimidiatus*	M	Rump	Yes	Yes	Yes	Yes
*R. flammigerus*	M	Rump	Yes	Yes	–	Yes
*R. f. icteronotus*	M	Rump	Yes	Yes	Yes	–
*R. melanogaster*	M	Upper throat	Yes	Yes	Yes	Yes
*R. melanogaster*	M	Lower throat	Yes	Yes	Yes	Yes
*R. nigrogularis*	M	Rump	Yes	Yes	–	Yes
*R. passerinii*	M	Rump	Yes	–	–	Yes
*R. sanguinolentis*	M	Rump	Yes	Yes	Yes	Yes

In contrast, male *Ramphocelus* tanagers exhibited wide variation in feather microstructure (Fig. 4, Table 1, Table S3, Fig. S8), including widely expanded barbs, oblong barbs, strap-shaped barbules (rather than cylindrical), and angled barbules that projected from the plane of the feather (Fig. 4E, Table 1). Among the angled barbules, we observed a dihedral morphology insuper black and velvet red feathers as described previously for super black plumages of *R. flammigerus*^29^*.* Two of these unusual male morphological traits, which we assess through optical simulations, deserve special mention (Fig. 4).

*Dihedral.* In the dark red crown of *R. carbo*, feathers have densely packed, strap-shaped barbules pointing upwards out of the plane of the feather to form a dihedral structure, while the central barb was shaped like a razor or narrow triangular prism (Fig. 4C). *Ramphocelus dimidiatus* shared the dihedral morphology present in *R. carbo* in its upper throat feathers, which are a velvet red color (Fig. S8). Both are reminiscent of the structurally absorbing “super black” feather morphology present in *Ramphocelus flammigerus*^29^. Similar morphology, to a lesser degree, was observed in *R. melanogaster* velvet red throat feathers and in red feathers of both male and female *R. sanguinolentis* (Fig. S8G,J)*.* To the human eye, dihedral feather structure generates a velvety appearance.

*Expanded barb.* Multiple species had expanded barbs, including the broad and roughly cylindrical barbs of the rump feathers of *R. flammigerus* (e.g., Fig. 4B,D, R*. nigrogularis*, and *R. passerinii*). These feathers also had flatter, strap-shaped barbules shorter in length than that of females but wider in width. Additionally, the rump and body feathers of *R. dimidiatus* are vivid red feathers with strong specular reflections; these feathers have a more extremely expanded central barb with a featureless surface (Fig. 4D). They also have upward-slanting, densely packed, strap-shaped barbules (Fig. 4D). Together, this generates (to the human eye) strong specular reflection at an angle, so that the bird shows flashes of white reflection when rotated in the hand.

A PCA of feather microstructure measurements demonstrated that males and female cluster separately and are not correlated (Fig. 4F; PC1 55.6%, PC2 20.8%; PGLS with microstructure PC1 scores: coefficient = -0.023, SE = 0.17, p = 0.90); a phylogenetic PCA was not possible to compare males and females, because such analyses can consider only one value per species, but we performed sex-specific phylogenetic PCAs of one patch per species and observed some clustering by clade for males (whole-body versus rumped, Fig. S6) and that females cluster tightly with the exception of outliers *R. sanguinolentis* and *R. bresilius,* both of which are females with wider-than-median barbs (Table 1). *R. sanguinolentis* is also the female with the most male-like scores in the regular PCA (Fig. 4F). Barb width, barbule width, barb oblongness, and interbarbule distance were primary axes along which males and females separated (Table S5). No species-specific or region-specific (e.g., rump feather versus chest feather) clustering was observed in these microstructural measurements (Fig. S6). In particular, it was important that we observed no region-specific variation in microstructure (i.e., rump feathers from one species did not cluster with rump feathers from other species).

### Male barb microstructure correlates significantly with plumage saturation

To test whether microstructures may influence plumage appearance, we used a phylogenetic generalized least squares model to assess whether male microstructure PC scores (Fig. 4) correlate with to plumage saturation (Fig. 2). We found that PC2 was a significant correlate of plumage saturation, whereby more oblong and wider barbules were associated with more saturated plumage (PC2 coefficient = 20.8, SE 6.79, p < 0.025; see Fig. 4).

### Optical simulations suggest that male-typical microstructures can enhance (i) blackness and (ii) color saturation

Using 2D finite-difference time-domain (FDTD) simulations of idealized feather cross-sections, we isolated the effect of two male-typical structure on reflectance: dihedral barbules and expanded, oblong barb. We selected these two features because they were associated with (i) male–female differences in color appearance within a species and (ii) within-bird color changes from bright, saturated red to dark, matte velvet red (Fig. S7).

We found that “dihedral” barbules^29^, projecting out of the plane of the feather and associated with super blacks and velvet reds in our study species, contribute to a lower reflectance (Fig. 6A). Total reflectance based on light-keratin interactions alone, without considering the contribution of melanin or carotenoids, drops from ~ 4% to 0.5% as barbules increase in angle from 0° to 80°. That is, the dihedral feather morphology in super black regions of these tanagers are made blacker in part due to structure. Likewise, the velvet red feathers in *R. carbo* and *R. dimidiatus* are made darker and more matte by melanin pigments in combination with dihedral barbule microstructure.

Further, we found that male-typical expanded feather barbs, which are wider than female barbs in 80% of species and more oblong than female barbs in 60% of our species (Table 1), substantially enhance optical power flow through the pigmented portion of the feather (Fig. 6B-C). In other words, this male-typical barb shape focuses light to cause greater light-pigment interactions, even though females and males across species have the same amount and types of pigment (Fig. 3). Therefore, the structure may contribute to a richer, more saturated color in the same manner as flower petals’ conical epidermal cells^36^. Interestingly, oblong and wide male barbs focus light more in particular regions within the feather barb (although they also increase optical power transmission generally across the whole barb, akin to flower petals^37^). Because the male-typical feathers focus light more on particular regions, it may be the case that pigments are not distributed evenly within the feather barb; further work with cross-sectional data is needed to investigate this idea.

We predicted that the ~ 30–45° angled barbules of many males, paired with an expanded barb, would further enhance color saturation, akin to angled flower petals causing a more saturated color^77^; simulations showed that the angled barbs contributed negligibly compared to the very large effect of an expanded barb. Across all sensitivity simulations (whole-feather, truncated feather, and feather with vacuole) and locations within the feather barb (side, top, and 45° angle) males had greater optical power transmission (Table S6; one-way paired t-test, p = 0.0021, mean of difference = -125.9, 95% CI = [-Inf, -64.2]).

**Figure 5:**
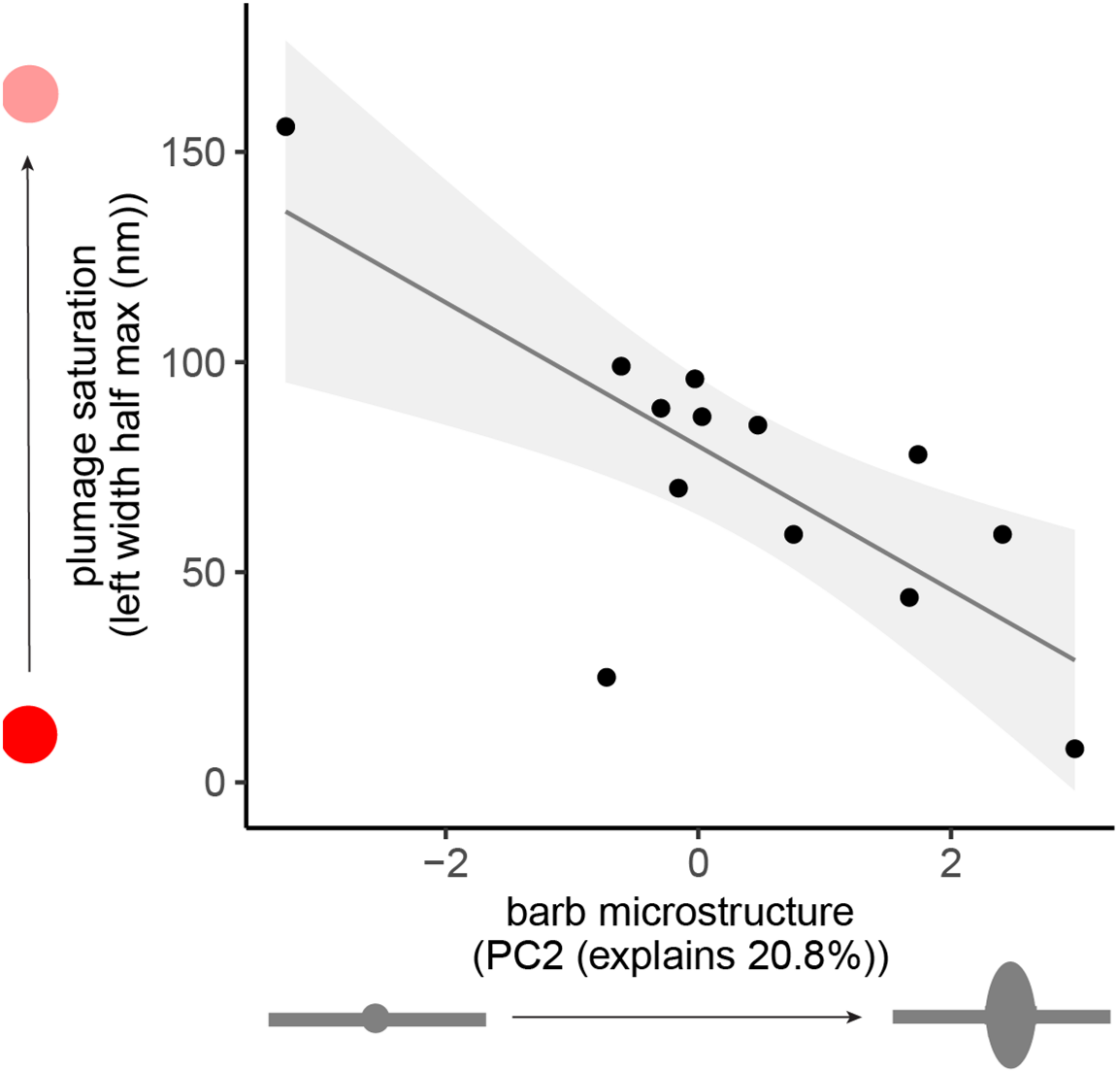
Male barb microstructure correlates significantly with plumage saturation. Wider, more oblong male barbs are correlated with more saturated plumage, as measured through left-width half-maximum of reflectance curve (see Fig. 2). We performed a PGLS with microstructure PC2 and PC1 scores as independent variables, and with plumage saturation as dependent variable (PC2 coefficient = 20.8, SE 6.79, p < 0.025; PC1 coefficient = 12.6, SE = 7.49; p = 0.1361).

It is important to note that these simulations represent idealized feather cross-sections, not real cross-sectional data. Our simulations provide proof-of-concept that expanded, oblong barbs in males focus light differently that simple barbs in females, enhancing color saturation; our simulation results are further buttressed by the observation that microstructural measurements are significantly correlated with color saturation (Fig. 5). However, further work to identify the specific locations of pigment, vacuoles, and internal nanostructures is needed to fully characterize these feathers.

### Spectral curve-fitting suggests that male–female color differences result from both (i) melanin in females and (ii) microstructures in males

**Figure 6:**
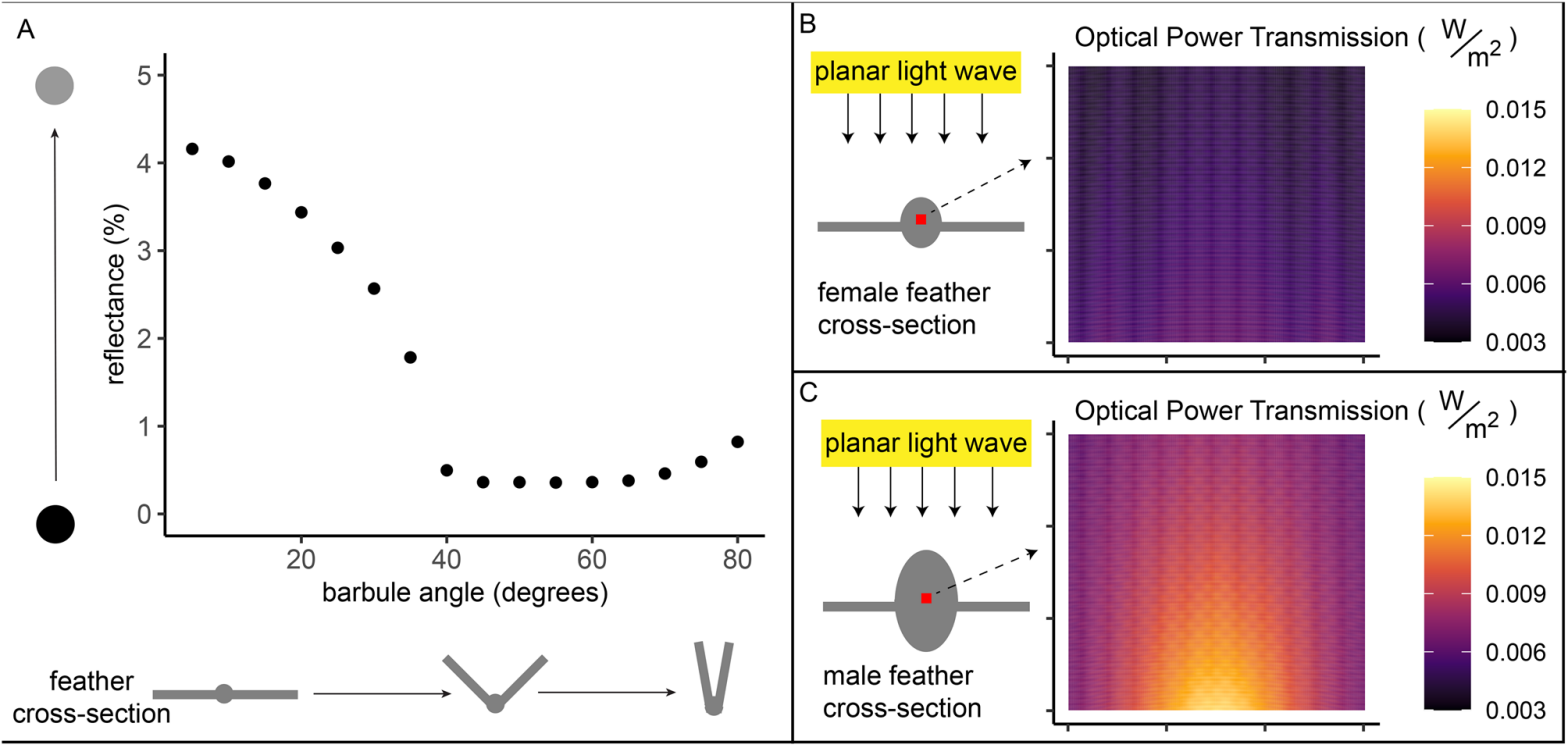
Optical simulations show that male-typical microstructures can (i) enhance blackness and (ii) increase color saturation. All simulations are finite-difference time-domain (FDTD) simulations on 2D idealized feather cross-sections, simulating structural effects only without accounting for pigmentary absorption. (**A**) A male-typical black feather, with angled barbules, has structurally enhanced blackness compared to a female-typical feather with flat barbules. As the barbule angle increases out of the plane of the feather, percent reflectance arising from the keratin structure alone decreases from 4% to 0.5%. We did not simulate absorption effects of melanin, but instead we isolated the effect of structure alone. (**B**) For a female-typical colored feather, the amount of light passing through the pigmented barb (optical power transmission through red square) totals 452.4 W/m^2^. (**C**) For a male-typical colored feather, an oblong, expanded central barb increases the amount of light passing through the pigmented barb (optical power transmission through red square; totaling 788.1 W/m^2^). This enhanced power transmission enhances pigmentary activity for the same amount of incident light. For (**A**), each dot is the average of 3 simulations (and an error bar is plotted although not visible because all standard deviations were < 0.035). For (**B,C**), optical power transmission is shown for 700 nm light, at the center of a barb, and for a single simulation, but males have greater optical power transmission than females over all wavelengths, four different locations within the feather barb, and three simulation types (see methods; Table S6; one-way paired t-test, p = 0.0021, mean of difference = -125.9, 95% CI = [-Inf, -64.2]).

We used curve-fitting as a preliminary test of whether (i) melanin in females and/or (ii) the optical impacts of male microstructures, as identified by our FDTD simulations (Fig. 6), could explain male–female color differences (details in Supplemental Methods).

**Figure 7:**
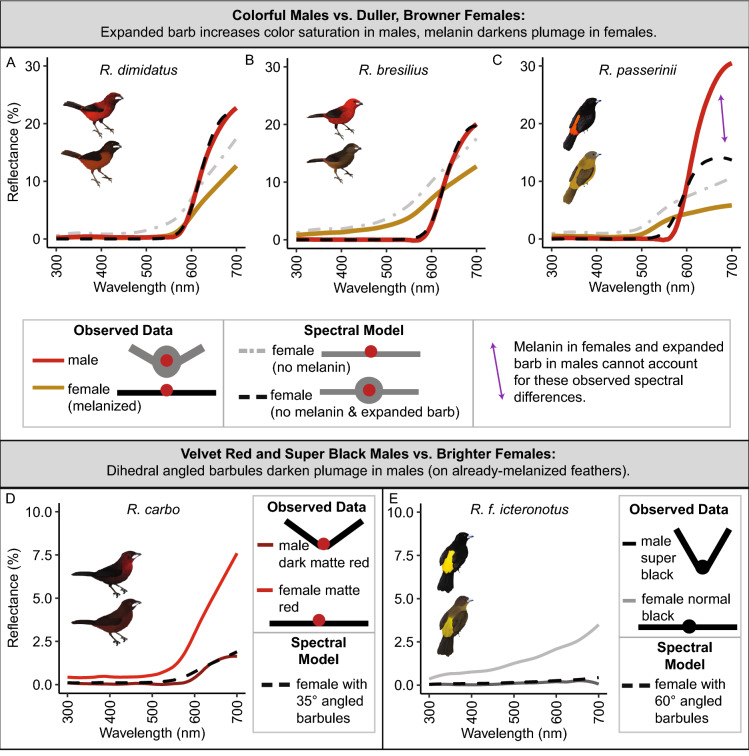
Curve-fitting with reflectance spectra suggests that male–female color differences are attributable to (i) melanin in females and (ii) microstructures in males. We manipulated real reflectance spectra of females to assess the role of melanin and microstructures in male–female differences (Supplementary Methods). We compared (**A**–**C**) colorful, saturated males to duller, browner female and (**D**–**E**) velvet red and super black males to relatively brighter females. (**A**) Reflectance spectra of a female *R. dimidiatus* rump (orange line), minus the effect of melanin (grey dashed line), and plus the predicted saturation increase due to an oblong expanded barb (dashed black line) matches well with observed reflectance spectra of a male *R. dimidiatus* rump (red line). Sigmoid curve parameters are a_female_ = 0.02, a_male_ = 0.06, b = 620, c = 22. (**B**) Reflectance spectra of a female *R. bresilius* rump (orange line), minus the effect of melanin (grey dashed line), and plus the predicted saturation increase due to an expanded barb (dashed black line) matches well with observed reflectance spectra of a male *R. bresilius* rump (red line). Parameters are a_female_ = 0.02, a_male_ = 0.06, b = 630, c = 20. (**C**) Reflectance spectra of a female *R. passerinii* rump (orange line), minus the effect of melanin (grey dashed line), and plus the predicted saturation increase of an expanded barb (dashed black line), is a poor match (purple arrow) for the observed reflectance spectra of a male *R. passerinii* rump (red line). Parameters are a_female_ = 0.02, a_male_ = 0.06, b = 590, c = 14. (**D**) Reflectance spectra of female *R. carbo* dark red breast (red line), divided by 4 to indicate structural contribution of dihedral barbules at a ~ 35° angle (dashed black line), matches well to observed reflectance spectra of *R. carbo* dark matte red breast (dark red line). (**E**) Reflectance spectra of female *R. f. icteronotus* black back (grey line), divided by 8 to indicate structural contribution of dihedral barbules at a ~ 60° angle (dashed black line), matches well to observed reflectance spectra of male *R. f. icteronotus* super black back (black line). Divisors for each barbule angle can be found in Fig. 6A. Artwork in male silhouettes credit Gabriel Ugueto; female silhouettes are modified by Allison Shultz from original art by Gabriel Ugueto. For information about melanin presence, see Supplementary Information.

We found that both melanin and microstructures were required to explain male–female differences in colorful red and orange plumage (Fig. 7A–C). For both *R. dimidiatus* and *R. bresilius*, curve simulating “female minus melanin plus expanded barb” matched well with the observed reflectance spectra of male rumps (Hausdorff distances for male versus simulated female-melanin + structure were 0.7 (*R. bresilius*; compared to 7.5 before manipulation) and 1.9 (*R. dimidiatus*; compared to 10.8 before manipulation). However, for *R. passerinii,* the simulated curve (dashed black line Fig. 7C) was a poor match.

We found that microstructure alone—angled barbules—was sufficient to explain differences between (i) velvet red male, and brighter red female, plumages and (ii) super black male and normal black female plumages (Fig. 7D). For velvet red male R. carbo plumage, the corresponding female reflectance spectra divided by 4 matches closely to the male (Hausdorff distance 0.4), corresponding to barbules at an angle of ~ 35° (Fig. 6A). Likewise, for super black male R. f. icteronotus, female plumage divided by 8 matches well to the super black male (Hausdorff distance 0.05), which corresponds to barbules at an angle of ~ 60° (Fig. 6A).

### Velvet red (versus bright red) within a bird arises from microstructure and melanin, not change in carotenoids

To better assess the relative contribution of microstructure and pigment, we compared within-bird pigment profiles of shiny saturated red patches versus dark velvet red patches for both *R. melanogaster* and *R. dimidiatus* (Fig. S7). For each bird, these two patches had significantly correlated pigment profiles at equal levels for *R. melanogaster* and with slightly more pigment in the bright red region for *R. dimidiatus* (Fig. S7A,E; Regression output for *R. melanogaster*: slope = 0.99, SE = 0.051, R^2^ = 0.93, p < 0.0005. Regression output for *R. dimidiatus*: slope = 0.76, SE = 0.061, R^2^ = 0.85, p < 0.0005.) However, the SEM photos revealed large differences in feather microstructure between dark and bright patches (Fig. S2B,C,F,G). Expanded barbs were associated with bright saturated color while vertically-angled or dihedral barbules were associated with velvet red color. Importantly, digital light microscopy showed melanized barbules in the velvet red feathers of *R. dimidiatus*, suggesting that melanin is present in velvet red plumages.

## Discussion

### Summary

Why are so many birds colorful? Our study directly addressed a proximate, physical “why” by showing that microstructures significantly enhance the red, orange, and yellow colors in male carotenoid plumages. Across species, colorful male and significantly drabber female *Ramphocelus* (Figs. 1–2) have significantly correlated carotenoid pigment profiles at a female:male ratio of 0.95 (Fig. 3). However, unusual microstructures in male (but not female) feathers augment male appearance in two ways (Figs. 4–7). First, wider, more oblong barbs in males create a more vivid, saturated color from structure alone without requiring more pigment. Second, dihedral barbules in male feathers produce (i) “velvet red” feathers adjacent to brilliantly reflective beaks and (ii) “super black” near colorful patches. We observe that microstructures contribute significantly to color signal appearance in males, rather than pigment alone.

But *why* are so many birds colorful? A deeper evolutionary “why” asks for the history of selective forces that produce colorful patterns and ornaments. Carotenoid-based coloration has often been invoked as a “textbook”^6^ or “classic”^78^ example of honest signaling, whereby the amount and types of pigments are thought to be costly or indicative of metabolic function^6–8,20,79^. In contrast to straightforward honest signaling theory, we propose that males have been selected to amplify their appearance by microstructural enhancers that are not themselves necessarily linked to quality. Our results show that pigments alone are insufficient to explain appearance, and therefore any theory of sexual selection (honest signaling or otherwise) must also explain the diversity of color-enhancing microstructures in males. Diverse microstructures in males suggest an evolutionary arms race between female preference and male appearance, which we term the “proxy treadmill”^80^. Our empirical results support past work by Fisher and others who predicted that one sex could evolve deceptive amplifiers in mate choice through an arms-race dynamic^3,81–84^. We discuss the proxy treadmill and other possible interpretations below.

### Pigments: males and females have concordant carotenoids

Unexpectedly, males and females across species had significantly correlated carotenoid profiles in feathers with a female:male ratio of 0.95 (Fig. 3; MCMC glmm). Because our LCMS method does not allow direct comparisons between different pigments, we confirmed that male and female pigments do not differ significantly using a within-pigment-family phylogenetic paired t-test. These results are exploratory, across-species, findings (rather than precise within-species quantifications). See further discussion in Limitations section below.

If carotenoids were present in feathers as an honest (because costly) signal, it would be surprising to see the same levels in females (the choosing sex) as in males (the displaying sex). It is difficult to argue that the presence of carotenoids in feathers is costly, is favored in males because it is costly, but females who gain no benefit therefrom have been unable to exclude carotenoids from their feathers (and indeed deposit melanin to dampen the color; Fig. S4). However, it is still possible that carotenoids are an index of metabolic function or that sexual selection is acting on both males and females.

It is worth dedicating a few sentences to the significance of dietary versus metabolized carotenoids. Carotenoids are consumed in a yellow (dietary) form and then metabolized within vertebrate bodies to become redder in color; red, not yellow, carotenoids seem to drive correlations between male appearance and health^6^. Oddly, it appears that *R. f. icteronotus* and *R. flammigerus* evolved to use dietary carotenoids (yellow  and orange) from a likely ancestral state of metabolized (red) carotenoids (Fig. 3, Fig. S1;^59^). Yellow-to-red transitions, but not red-to-yellow, are common in model clades (fringillid finches^85^; new world blackbirds^86^). The possible reversion from red to yellow and orange documented herein is rare and worth further study; perhaps *Ramphocelus* males were not selected to display metabolically indicative or costly traits. In a hybrid zone, Morales-Rozo and colleagues demonstrate that the yellow *R. f. icteronotus* outcompetes the orange *R. flammigerus* in areas where both male phenotypes occur (i.e., where females have a choice^87^). Perhaps females prefer a less-elaborate, less-costly trait—as predicted by Hill^81^ for an arbitrary rather than honest signal. Further research should be done on the behavioral ecology of these fascinating tanagers to clarify female preferences and social interactions.

### Structures: microstructures amplify plumage appearance in males

Feathers in *Ramphocelus* males, but not females, vary remarkably in microscopic structure (Table 1, Fig. 4, Fig. S8). Taken together, our data (Fig. 5), optical simulations (Fig. 6), and post-hoc curve fitting (Fig. 7) suggest that microstructures cause males to have more saturated colors than females, darker blacks than females, and—in some species—striking velvet red coloration.

**Figure 8:**
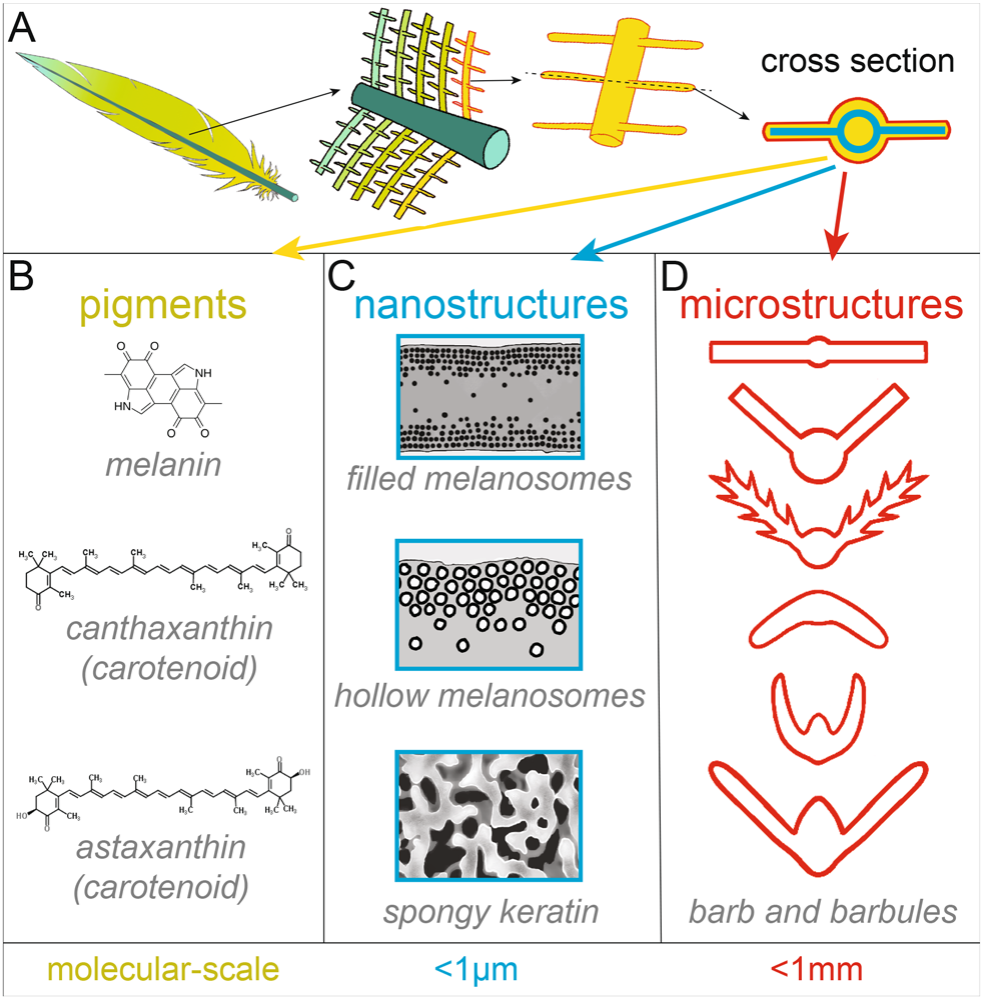
Feather color is produced by three interacting features: pigments, nanostructures, and microstructures. This schematic is a simplified illustration of the major physical causes of feather color; plumages are immensely variable. (**A**) From left to right, we show (i) a feather, (ii) a zoomed-in view of the rachis, barb, and barbules; (iii) the barb and barbules; (iv) an idealized cross section of the central barb and barbules. (**B**) At the molecular scale, pigments such as melanin, canthaxanthin, and astaxanthin produce colors^22,114,115^. (**C**) At the sub-micrometer scale (< 1 µm), nanostructures^116^ such as filled melanosomes^33^, hollow melanosomes^34^, and spongy keratin matrices^35^ produce iridescent colors and blue colors. (**D**) Between 1 µm and 1 mm, microstructures of the barb and barbules affect pigment saturation, gloss, and brightness^23–31^. All illustrations in this figure are by A. Kimber.

Oblong, expanded barbs in males increase the optical power transmitted through the pigmented barb (Fig. 6B-C): more light interacts with the same amount of pigment to generate a more saturated, vivid color (Figs. 1, 2). This is analogous to conical epidermal cells in flower petals, which enhance light transmission through the whole petal but enhance it to a greater degree in the pigmented region^37^. Further cross-sectional studies of feather pigment location—and potentially impactful nanostructures (e.g. Fig. 8C)—would be invaluable.

Upward-angled barbs in a dihedral arrangement reduce total reflectance from a plumage (Fig. 6A) to make super black color (if paired with melanin) or velvet red color (if paired with carotenoids; see *R. carbo* Fig. 1D with velvet red adjacent to a brilliant silver beak). Super black color is an optical illusion which enhances the perceived brilliance of adjacent colors^28,29,40,88–90^, thereby potentially amplifying male appearance. The velvet red color in *Ramphocelus* tanagers calls to mind mourning cloak butterflies *Nymphalis antiopa*. It is intriguing to speculate that the structures causing super black in butterflies^91^ also underly this velvet red color in *Nymphalis* when combined with red (rather than black) pigment.

Research on microstructural variation in colorful displays, including sex differences, is expanding rapidly^24,26,28–31,92^. To gain more insight into evolutionary dynamics, we require a complete understanding of the physical basis of color. This means accounting for the optical effects of microstructures in addition to the traditionally studied pigments and nanostructures^93^ (Fig. 8).

### The proxy treadmill: honest signals can be gamed, causing trait elaboration

Why did male feathers evolve elaborate microstructures? If female choice is a significant driver of male plumage, then males will be selected to produce female-preferred signals whether this is by changes to the chemical composition or microstructure of their feathers. From this perspective, the evolutionary dynamics of female preferences and male traits has aspects of an arms race between the conflicting interests of the sexes^80–82,84,94,95^. Females establish tests of quality and males are selected to pass these tests whether by ‘honest’ displays or ‘gaming’ the test. Rather than eating and metabolizing more carotenoids to honestly signal a more saturated red^5–9^ males could use microstructural amplifiers to make their plumage appear a more saturated red. Following substantial past work^80–84,94,96^, we suggest that males, under intense selection to satisfy testing criteria by choosy females, evolve “amplifiers” to honest signals (in this case, microstructures that enhance light-pigment interactions).

Perhaps red carotenoid-based colors were favored by female selection as an index of physiological health, but selection on males to deliver a saturated red signal by whatever means gradually diluted the information about male quality^81^. As males exaggerate their appearance, females are selected to develop additional quality tests to separate the wheat from the chaff (e.g., females may prefer even more saturated feathers or new, additional ornaments). Because females cannot unilaterally abandon a prior quality test without dooming their sons to being unattractive, male ornaments may pile atop one another, potentially causing extreme and elaborate signals. This is the proxy treadmill^80^: signal traits become exaggerated as proxies of quality are continuously modified or replaced (because examinees are under strong selective pressure to inflate their apparent quality, thus devaluing any given proxy).

Males of many taxa find ways to amplify their carotenoid signals. Male guppies (*Poecilia reticulata*) have orange carotenoid-colored spots for sexual display. Recall that carotenoids must be eaten and metabolized rather than synthesized de novo, a commonly-cited reason supporting the idea that carotenoids are honest signals. Guppies synthesize (de novo) red pteridine pigments (drosopterins) similar in hue to carotenoids and include these pigments in their red spots. The authors suggest that male guppies use drosopterin pigments “in a manner that dilutes, but does not eliminate, the indicator value of carotenoid coloration”^78^. Greater flamingos (*Phoenicopterus roseus)*, a classic example of carotenoid-colored birds, amplify their plumage color by “applying cosmetics”: they secrete carotenoid-colored preen oils to coat their feathers, and do so more often during display times^97^. In these and other cases, it is difficult to test which elements of multicomponent signals are linked to quality (if any).

Beyond carotenoids, direct evidence for deceptive elements to honest signaling have been reported in many invertebrates^98–100^ and a few vertebrates^101–103^. Beyond mate choice, the analogous arena of embryo selection shows evidence of the proxy treadmill^80^. During human pregnancy, embryos “audition” for the role of a lifetime^104^ and must pass signaling “checkpoints” to be carried to term^105^. One measure of embryo quality, the signaling hormone chorionic gonadotropin, has seen an extreme inflation over evolutionary time with no clear functional significance^80^—reminiscent of the elaborate microstructures that underpin male *Ramphocelus* color. Goodhart’s Law in economics and the analogous Campbell’s Law describe this well-observed phenomenon: “when a measure becomes a target, it ceases to be a good measure”^106^.

In addition to our proposal—the proxy treadmill—sexual selection is complex and many selective forces likely interact to influence coloration, including predation risk^16,107^, intra-sex aggression^12–14,17^, honest signaling via the structures themselves^108–111^, arbitrary preferences^2^, and species recognition^1^. Perhaps in these tanagers, both sexes are subject to sexual selection for carotenoid colors, but with sex-dependent tradeoffs between naturally selected crypticity (higher in females) and sexually selected conspicuousness (higher in males).

### Limitations and conclusion

We would like to note limitations of this exploratory study which should be addressed in detailed follow-up work. First, we have data from only one male and one female of each of ten species; future work could include data from many males and many females per species in order to conduct detailed within-species comparisons. Second, we do not report cross-sectional data for our feathers, which could be achieved through transmission electron microscopy for detailed future work on optics. Third, we used a mechanical extraction procedure for the carotenoids, and it would be useful to conduct follow-up tests comparing this extraction procedure to pyridine extraction^112^. Fourth, we used LCMS to identify carotenoids with signal strength as a proxy for amount. The signal strengths cannot be compared directly between pigment families because carotenoids ionize differently depending on the molecular structure. Therefore, follow-up work could employ more tradition UV–Vis quantification. Fifth, we did not look for carotenoid esters, which can comprise a portion of feather pigments^53^; therefore future work could benefit from UV–Vis-MS analyses. Finally, more work is needed to understand which, if any, structural components of feather color are linked to quality (see^113^ for an analysis of carotenoids paired with white structural color; Shawkey et al. found that the structural color in this case is “probably not condition dependent”^113^).

Few studies quantify plumage signals in both males and females, but a comparative approach can advance our understanding of evolution^44^. More broadly, signals cannot be pigeonholed as purely honest, purely deceptive, or purely arising from one selective force. Individuals struggle to satisfy many competing selective pressures that vary over time and space, from avoiding predators to finding a mate. Nature is red in tooth, claw, and male tanager feathers.

## Supplementary Information

Supplementary Information 1.

Supplementary Information 2.

## Data Availability

All data is included as supplemental information. Source data are provided with this paper. We provide source data for Figs. 2–7 and supplemental Figures S1–S6.

## Code Availability

All code is included as supplemental information.
