# Supplementary material for: Microstructures amplify carotenoid plumage signals in tanagers
Source: Sci Rep. 2021 Apr 21;11:8582. doi: 10.1038/s41598-021-88106-w (PMC8060279; doi:10.1038/s41598-021-88106-w)
Supplement: Supplementary file 1 — Supplementary Information 1. [file 41598_2021_88106_MOESM1_ESM.docx]

*Supplementary Information for:*

**Microstructures amplify carotenoid plumage signals in tanagers**

**Authors:** Dakota E. McCoy^1*^, Allison J. Shultz^1,2,3,4^, Charles Vidoudez^5^, Emma van der Heide^1^, Jacqueline E. Dall^4^, Sunia A.Trauger^5^, David Haig^1^

*1. Department of Organismic and Evolutionary Biology, Harvard University, 26 Oxford Street, Cambridge, MA 02138*

*2. Informatics Group, Harvard University, 38 Oxford Street, Cambridge, MA 02138*

*3. Museum of Comparative Zoology, Harvard University, 26 Oxford Street, Cambridge, MA 02138*

*4. Ornithology Department, Natural History Museum of Los Angeles County, 900 Exposition Blvd, Los Angeles, CA 90007*

*5. Harvard Center for Mass Spectrometry, Harvard University, 52 Oxford Street (B2), Cambridge, MA 02138*

* corresponding author: [dakotamccoy@g.harvard.edu](mailto:dakotamccoy@g.harvard.edu)

Contents

[Supplementary Methods 4](#_Toc65874789)

[Melanin Identification (Spectral Assessment and Digital Light Microscopy) 4](#_Toc65874790)

[Curve-fitting with Reflectance Spectra to Quantify the Contributions of Melanin and Microstructure 4](#_Toc65874791)

[Figure S1: Ancestral state reconstructions of each carotenoid pigment family. 6](#_Toc65874792)

[Figure S2: Metabolic map of carotenoid pigments. 7](#_Toc65874793)

[Figure S3: LC-MS pigment characterization is repeatable across individuals within a species. 8](#_Toc65874794)

[Figure S4: Male and female carotenoid pigments summed by family. 9](#_Toc65874795)

[Figure S5: Melanin presence in feathers, revealed by (A) mathematical assessment and (B-E) digital light microscopy. 10](#_Toc65874796)

[Figure S6: Additional PCAs uphold main results. 12](#_Toc65874797)

[Figure S7: Within-bird patches 13](#_Toc65874798)

[Figure S8: Complete SEM Results 14](#_Toc65874799)

[A. Ramphocelus bresilius 14](#_Toc65874800)

[B. Ramphocelus carbo 14](#_Toc65874801)

[C. Ramphocelus passerinii costaricensis 15](#_Toc65874802)

[D. Ramphocelus dimidiatus 15](#_Toc65874803)

[E. Ramphocelus flammigerus 16](#_Toc65874804)

[F. Ramphocelus flammigerus icteronotus 17](#_Toc65874805)

[G. Ramphocelus melanogaster 18](#_Toc65874806)

[H. Ramphocelus nigrogularis 18](#_Toc65874807)

[I. Ramphocelus passerinii 19](#_Toc65874808)

[J. Ramphocelus sanguinolentus 19](#_Toc65874809)

[Table S1: Specimen details. 21](#_Toc65874810)

[Table S2: NCBI Accession Numbers. 22](#_Toc65874811)

[Table S3: Microstructural measurements 23](#_Toc65874812)

[Table S4: Pigment identification using LC-MS. 24](#_Toc65874813)

[Table S5: PCA Loadings for microstructure PCAs (normal and phylogenetic). 25](#_Toc65874814)

[Table S6: Optical power transmission results for simulations of oblong expanded barbs. 26](#_Toc65874815)

**Additional Supplemental Files, in zipped Supplemental_Files folder:**

- Data Files:
  - **Carotenoid_Pigments_Ramphocelus.csv** includes complete LCMS carotenoid pigment findings for all species
  - **Microstructure_SEM_Measurements_Ramphocelus.csv** includes complete SEM microstructural measurements for all 10 species
  - **Spectrophotometry_Ramphocelus.xlsx** includes complete spectrophotometry results for all 10 species, including 6 body regions per species measured both at 90° and 45° incident light.
  - **pigment_names.csv** includes naming conventions for the carotenoid pigments studied
- R Code Files:
  - **carotenoid_analyses.R**: code for carotenoid analyses
  - **spectrophotometry_analyses.R:** code for spectrophotometry analyses
  - **microstructure_analyses.R:** code for microstructural analyses
- Lumerical Code Files:
  - **Dihedral_Barbules_Angle_Sweep.lsf**: optical simulations of dihedral barbules
  - **Expanded_Feather_Barb_Female_vs_Male.lsf**: optical simulations of an expanded feather barb
- Phylogenetic Trees
  - **ramphocelus_rooted_ingrouponly.tree**: Rooted ingroup-only *Ramphocelus* tree generated for analyses.
  - **ramphocelus_rooted_ingrouponly_ultrametric.tree** Ultrametric, rooted, ingroup-only *Ramphocelus* tree generated for analyses.

# Supplementary Methods

## Melanin Identification (Spectral Assessment and Digital Light Microscopy)

In some cases, male and female birds differed perceptibly in not only saturation but also brightness and hue, which (unlike most changes in saturation) could be explained by the addition of black absorbing media such as melanin (Gilbert and Haeberli 2007). We looked for evidence of melanin in feathers by assessing spectral shape and using digital light microscopy.

It is possible to mathematically assess the shapes of spectral curves to distinguish carotenoids from melanins (Toral et al. 2008). Toral et al (2008) identified an equation that captures strong differences in spectral shape for carotenoids versus melanins:

*Equation S1* $D1= -0.464\lambda_{520} -2.092\lambda_{640}+4.493\lambda_{660} -2.214\lambda_{740}+7.851$

D1 < 0 indicates melanins are present while D1 > 0 indicates carotenoids are present. The value of D1 itself is related to color saturation and the sigmoidal nature of the reflectance curve; it cannot be used to quantify the relative presences of melanin and carotenoid in a plumage, but it can usefully distinguish plumages that are primarily melanin-colored versus primarily carotenoid-colored. It could not necessarily identify a female bird primarily colored by carotenoids that also has deposited melanins into the feathers.

To supplement the results of this mathematical assessment, feathers were photographed at 100X using a Keyence V-5000 digital microscope with a white LED light source; we took high-resolution images of 8 feathers—4 each of males and 4 each of females of the same species—to consider the presence and location of melanin in the feathers. During the microscope setup, we white balanced the camera based on the white stage holding the feather, and minimal image processing was done within the Keyence software.

## Curve-fitting with Reflectance Spectra to Quantify the Contributions of Melanin and Microstructure

Our goal with the following methods was to assess the relative contributions of microstructure and melanin to male-female plumage differences. We adopted a curve-fitting approach whereby we manipulated the parameters of reflectance spectra of female plumages. In doing so, we could approximate the contributions of melanin—which changes *brightness* *and* *hue*—versus microstructures: (i) oblong, expanded barbs are predicted to change color *saturation* and (ii) dihedral barbules are predicted to change color *brightness.*

**Colorful Males vs. Duller, Browner Females.** We manipulated target spectra from *R. dimidiatus, R. bresilius*, and *R. passerinii* to investigate whether melanin, microstructures, or both explained male-female differences*.* First, to simulate the removal of melanin, we subtracted the reflectance spectra of the melanized back of female *R. costaricensis* from these three target spectra. Second, to simulate the addition of an oblong, expanded barb, we modeled each reflectance spectrum with a logistic function. Specifically, we modified parameters of a sigmoidal function to fit the curve of the target spectra. Carotenoid coloration generates a sigmoidal reflectance curve with parameters that vary based on saturation, hue, and brightness (Toral et al., 2008). The sigmoidal function with parameters *a,b*, and *c* is the function

*Equation S2*  $y= \frac{c}{1+e^{-a(x-b)}}$

where *a* is the slope, *b* is the x-intercept at sigmoid midpoint (steepest slope), and *c* is the upper asymptote (maximum y value). For our purposes, *a* represents pigment saturation (the higher the value of *a*, the steeper the reflectance curve, and the more saturated the color) and varies between males and females, while *b* and *c* describe the shape of the curve and overall feather brightness (thus, *b* and *c* should be held constant between males and females). To identify initial parameters *a, b, and c*, we fit a sigmoidal curve to the reflectance spectrum of female plumage minus the contribution of melanin. Then, we multiplied *a* by 3 to generate a more saturated feather color (while keeping *b* and *c* constant). We set a_male_ = 0.06 (all three fitted sigmoid functions to female-minus-melanin curves had a slope parameter a_female_=0.02).We generated a more saturated feather color to simulate the effect of male microstructure, because expanded oblong barbs increase optical power transmission—a cause of color saturation. We chose the multiplier of 3 through curve-fitting: specifically, we stepwise increased the multiplier starting with 2 until we found the best-fit curve (based on the Hausdorff distance; see below). We used the function sigmoid from the R package pracma (Borchers, 2013).

**Velvet Red and Super Black Males vs. Brighter Females.** To compare super black male plumages to normal black female plumages, and velvet red male plumages to brighter red female plumages, we manipulated target spectra from *R. carbo* and *R. f. icteronotus*. To assess whether microstructure alone could account for the differences in reflection, we simulated the brightness decrease associated with dihedral angled barbules. Dihedral barbule microstructures multiply scatter light, increasing the total amount of light transmitted into the feathers. Reflectance curves of such plumages have a spectral shape similar to that of a similarly pigmented feather with no microstructural enhancement divided by a factor of 3–12 (McCoy & Prum, 2019). We therefore divided the female reflectance spectra by integers beginning with 3 and increasing until we found the best-fit curve (a divisor of 4 for *R. carbo* dark matte red and 9 for *R. icteronotus* super black). We could then calculate the barbule angle using the results of our optical simulations (i.e., for what barbule angle would we expect a divisor of 4 to explain the difference between male and female feathers?). Finally, we compared the discovered barbule angles to real images of the feather microstructures.

To provide a quantitative score of all comparisons between male reflectance curves and simulated reflectance curves, we calculated the Hausdorff distance-- for (i) real female versus real male curve and (ii) real male versus manipulated female curve-- using function hausdorff.dist in R package pracma (Borchers, 2013).

# Figure S1: Ancestral state reconstructions of each carotenoid pigment family.


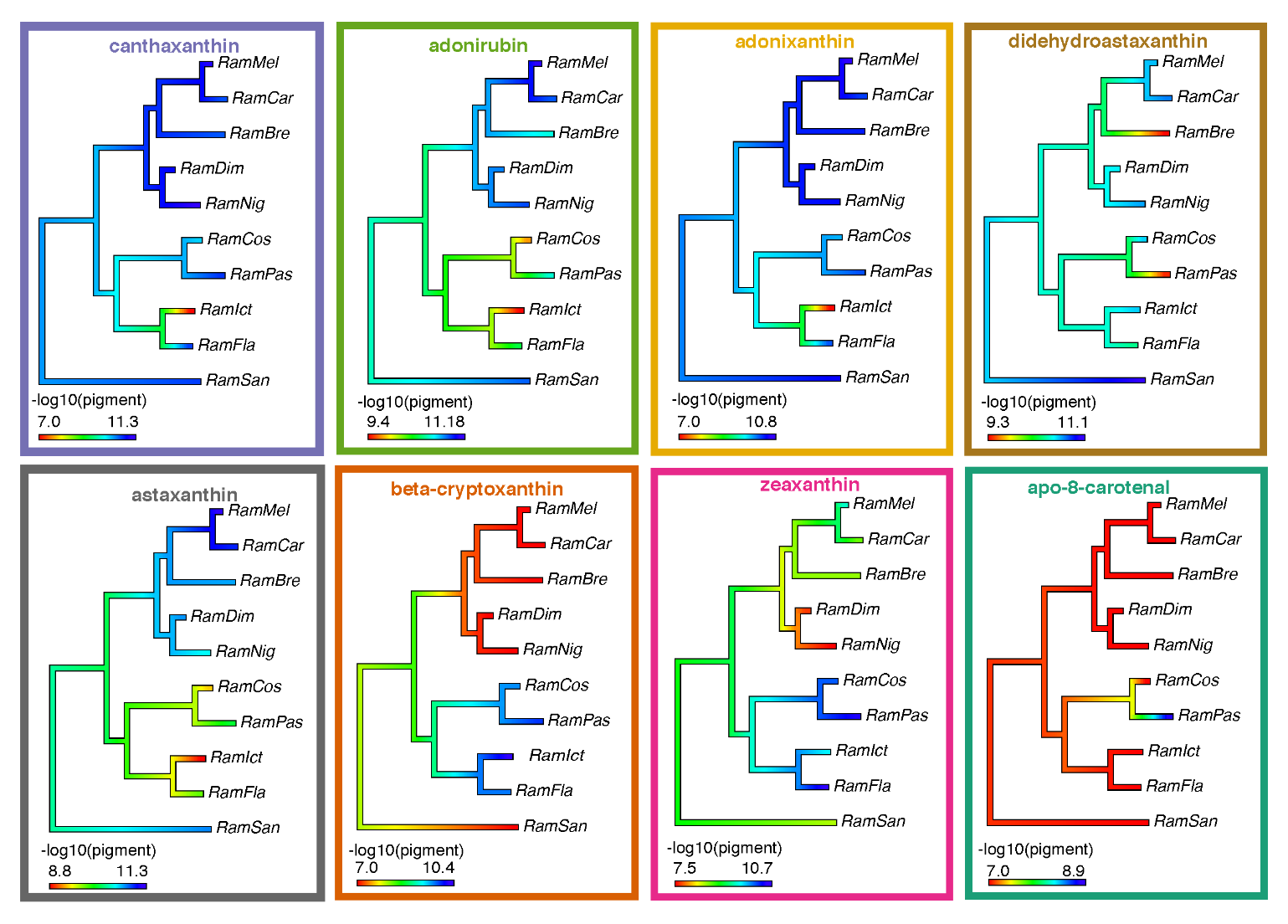


**Figure S1:** **Ancestral state reconstructions of each carotenoid pigment family.** We summed and log-transformed all isoforms within each pigment family, and then estimated ancestral states for each pigment.

# Figure S2: Metabolic map of carotenoid pigments.

**
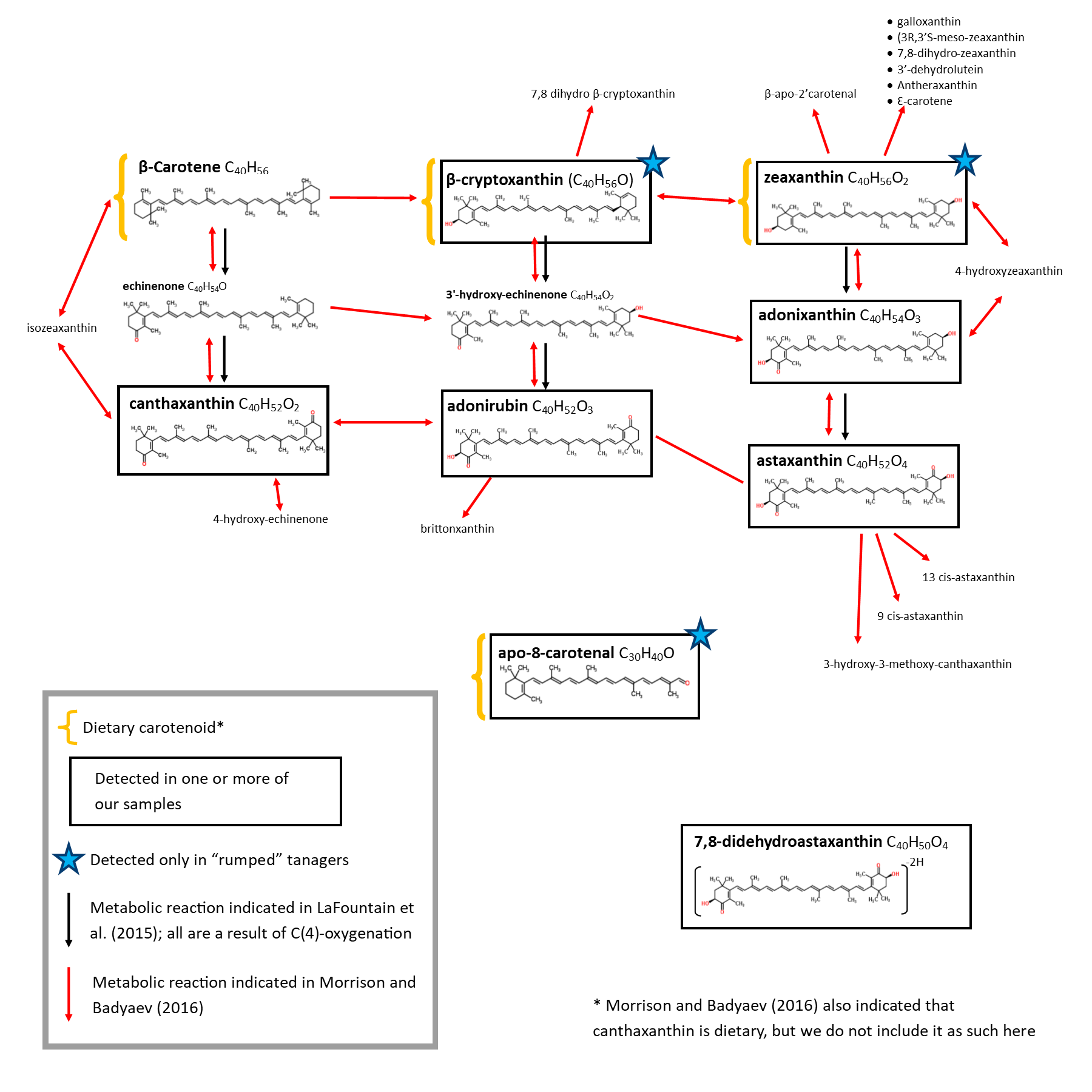
**

**Figure S2: Metabolic map of carotenoid pigments.** Based upon metabolic maps in the literature, we mapped our detected pigment families (in black boxes) into a metabolic network to demonstrate the presumed relationships between identified pigments. Apo-8-carotenal and didehydroastaxanthin could not be incorporated into the map and thus are shown separately.

# Figure S3: LC-MS pigment characterization is repeatable across individuals within a species.

*
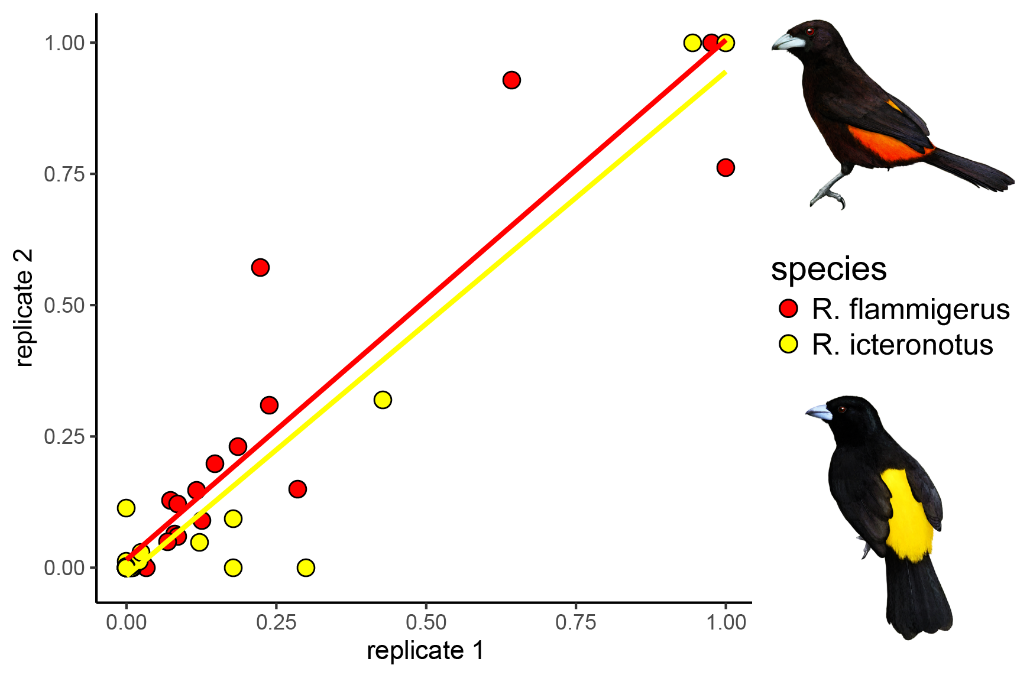
*

**Figure S3**: **LC-MS pigment characterization is consistent across individuals within a species.** Pigment profiles for 2 individuals per species of *R. f. icteronorus* and *R. flammigerus* demonstrate significant within-species correlations (linear trend line plotted for each). All values are normalized within an individual by feather weight and normalized such that the maximum signal was set to 1. Linear regression output for *R. flammigerus*: slope = 0.88, SE = 0.066, R^2^ = 0.87, p < 0.0005. Linear regression output for *R. f. icteronotus*: slope = 0.96, SE = 0.054, R^2^ = 0.92, p < 0.0005. Artwork in bird silhouettes credit Gabriel Ugueto.

# Figure S4: Male and female carotenoid pigments summed by family.

*
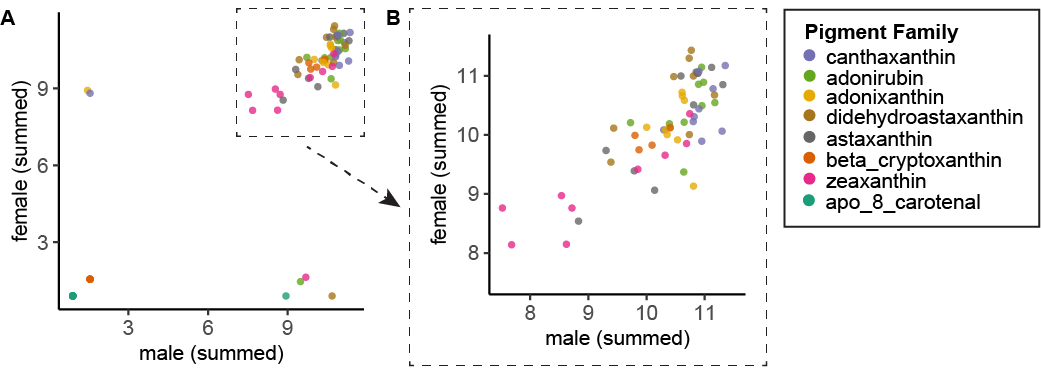
*

**Figure S4: Male and female carotenoid pigments summed by family. A.** Male versus female carotenoid pigments summed by pigment family; each point represents the signal strength (a proxy for presence and amount) of a given pigment family in both a male and female of one species. All values are log-transformed and normalized. Male and female pigment profiles summed by family are significantly correlated (MCMCglmm model when pigments were summed by family, posterior mean = 0.74, 95% CI = [0.52, 0.96], N_effective_ = 1800, p < 0.001, DIC = 377.9 ). **C.** Zoom in on male versus female carotenoid pigments as shown in **B** but, for visualization, restricted to points where male and female values were both greater than 0. For the un-summed data see Figure 3 in main manuscript.

# Figure S5: Melanin presence in feathers, revealed by (A) mathematical assessment and (B-E) digital light microscopy.

*
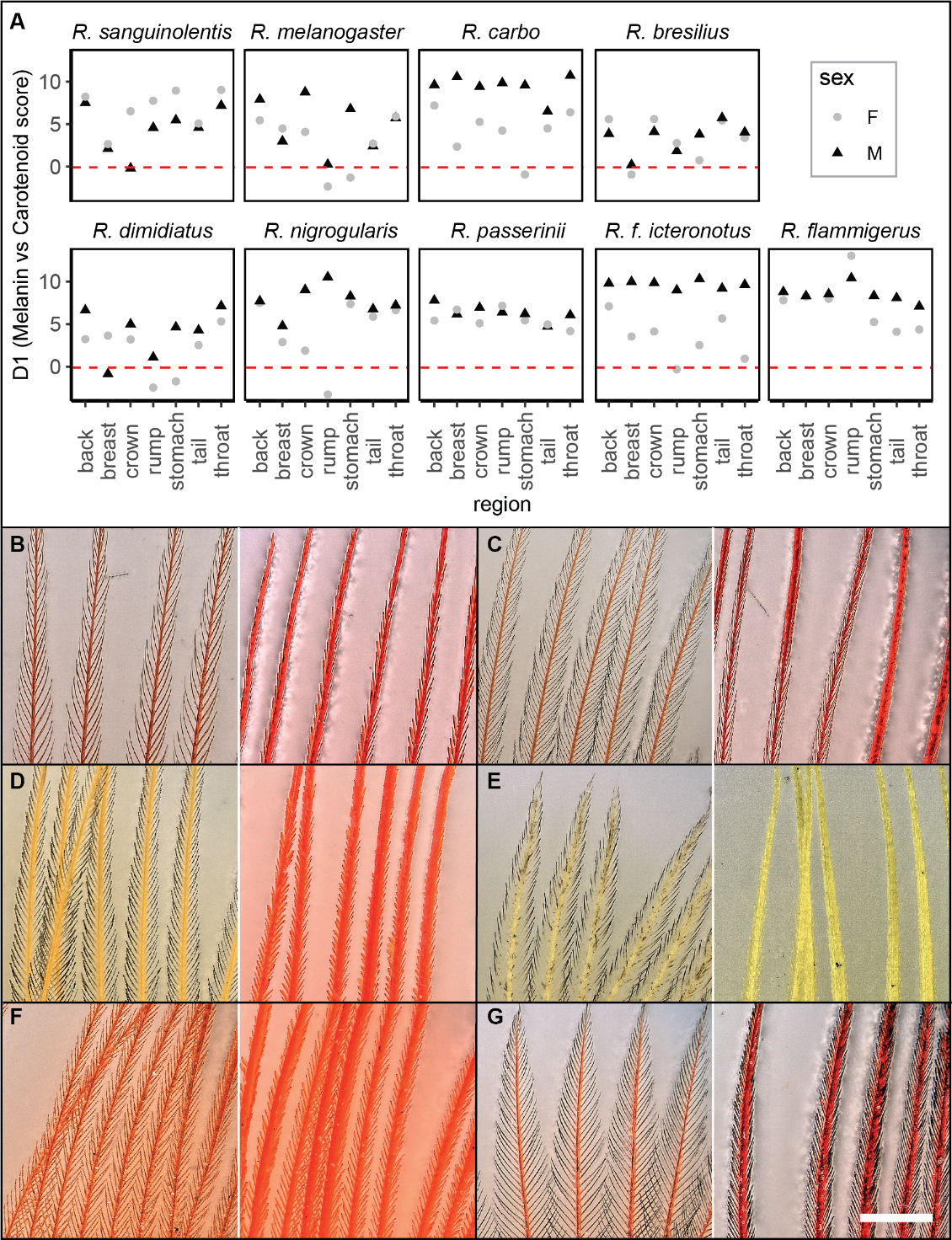
*

**Figure S5:** **Melanin presence in feathers, revealed by (A) mathematical assessment and (B-G) microscopy. A:** Using the equation from Toral et al. (2008) to distinguish melanin reflectance curves from carotenoid-rich reflectance curves (Equation 1), we found D1 scores less than 0 (below the red dashed line), which indicate melanin, in 7 plumage regions from females (*R. nigrogularis* rump, *R. dimidiatus* rump and stomach, *R. melanogaster* rump and stomach, *R. bresilius* breast, *R. carbo* stomach, and *R. f. icteronotus* rump. ) and 2 from males (*R. sanguinolentis* crown, *R. dimidiatus* breast). Microscopy of colored feathers from six species indicated that **B-E** for four species colorful female feathers had melanized barbules but males did not, **F** for one neither sex did**,** and **G** for velvet feathers both sexes did. **B:** *R. dimidiatus* rump feathers from female (left) and male (right). **C:** *R. bresilius* rump feathers from female (left) and male (right). **D:** *R. passerinii* rump feathers from female (left) and male (right). **E:** *R. f. icteronotus* breast feather from female (left) and rump feather fmro male (right). **F.** *R. flammigerus* rump feather from female (left) and male (right) **G.** *R. carbo* dark red back feathers from female (left) and male (right). Scale bar is 400 µm; applies to all panels B-G.­ In panels B-G, barbs are the central shaft running in the Y direction (bottom to top) while barbules emerge at an angle from the barbs.”

# Figure S6: Additional PCAs uphold main results.


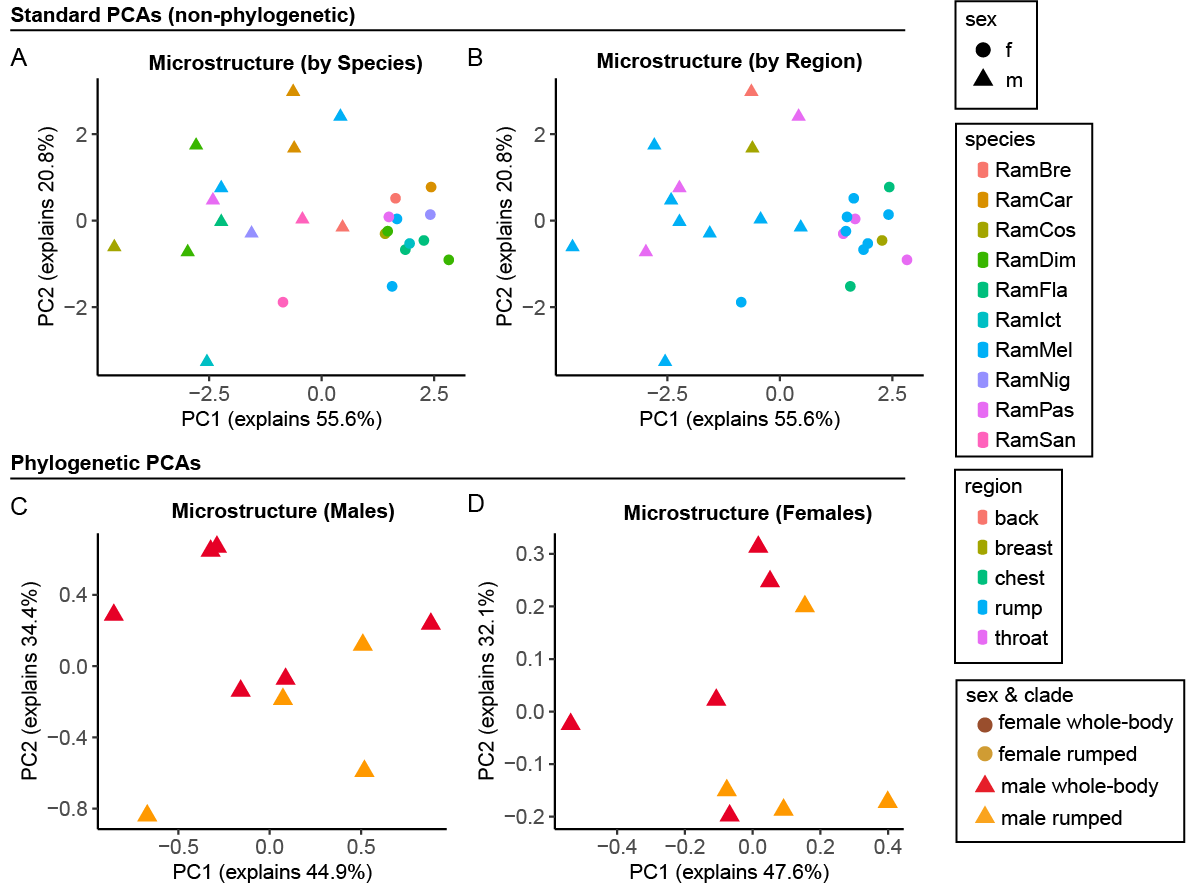


**Figure S6: Additional PCAs uphold main results.** A: Non-phylogenetic PCA of microstructural measurements for all measured feathers, males and females, grouped by species. B: Non-phylogenetic PCA of all microstructural measurements from all measured feathers grouped by region. C. Phylogenetic PCA of microstructural measurements for males (one feather per male per species). D: Phylogenetic PCA of microstructural measurements for females (one feather per female per species). For species where we had to select only one patch, we chose the following: Females: *R. melanoga*ster throat, *R. dimidiatus* rump, *R. flammigerus* rump, *R. passerinii* rump. Males: *R. dimidiatus* rump, *R. carbo* back.

# Figure S7: Within-bird patches

**
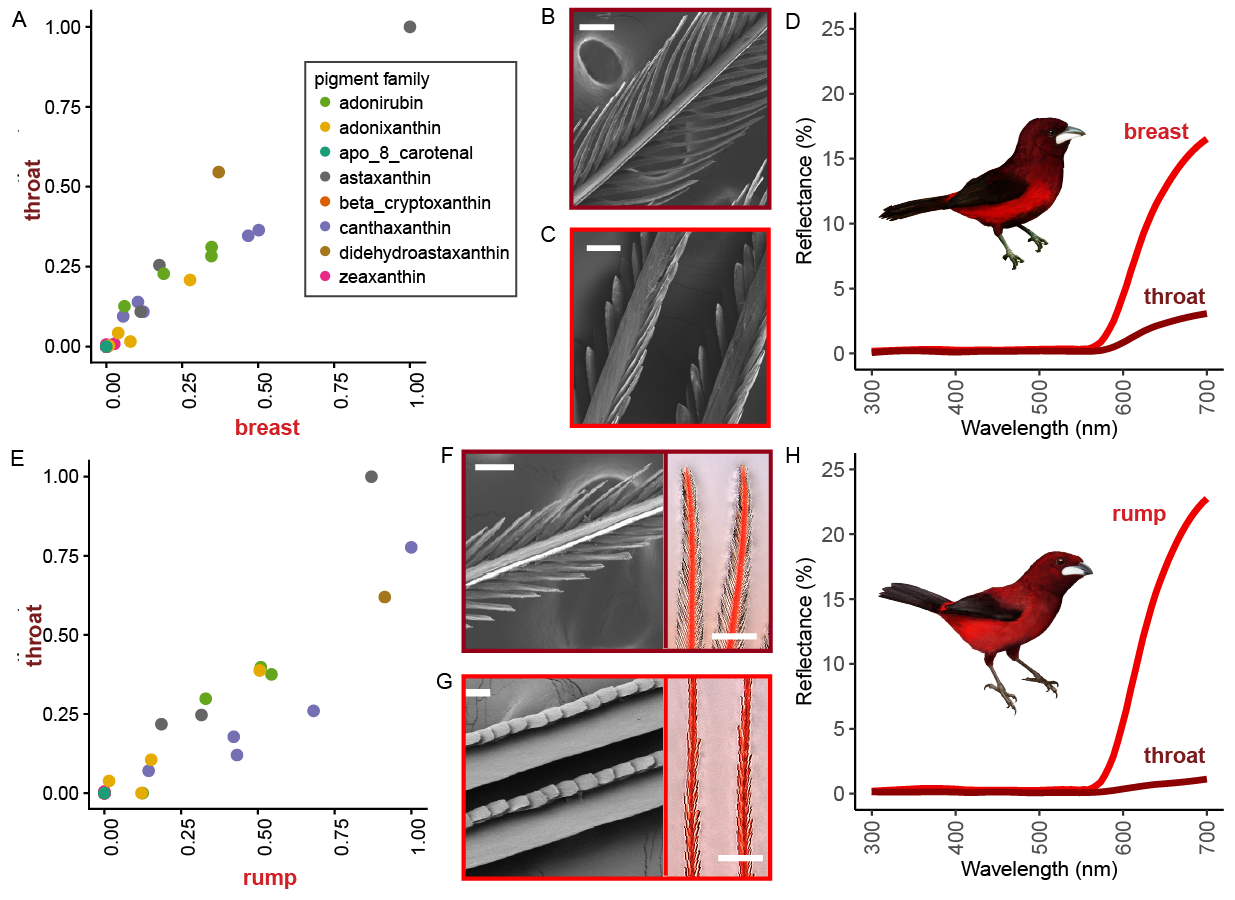
**

**Figure S7: Microstructure and melanized barbules, not carotenoids, differ between bright and dark red patches within a bird**. **A:** *R. melanogaster* velvet red throat versus brighter red breast. Pigments are highly correlated between regions: slope = 0.99, SE = 0.051, R^2^ = 0.93, p < 0.0005. **B-C:** *R. melanogaster* with SEM images of dark red throat feather (top) compared to brighter red lower throat feather (bottom). **D:** Reflectance spectra for *R. melanogaster* dark red throat feather and bright red breast feather. **E:** Pigment profiles of *R. dimidiatus* dark velvet red throat versus bright red rump, where each point represents the signal strength for one pigment molecule. Pigments are highly correlated between regions: slope = 0.76, SE = 0.061, R^2^ = 0.85, p < 0.0005. **F-G:** *R. dimidiatus* with SEM (left) and digital light microscopy (right) images of **F** dark red throat feather compared to **G** bright red rump feather. Digital light microscope images suggest melanized barbules in dark red throat. **H:** Reflectance spectra for *R. dimidiatus* dark red throat feather and bright red rump feather. All values are normalized such that the largest value (strongest signal) was set equal to one. Scale bars are 50 µm for SEM images and 300 µm for microscope images. Artwork in bird silhouettes credit Gabriel Ugueto.

# Figure S8: Complete SEM Results

## A. Ramphocelus bresilius

Female rump


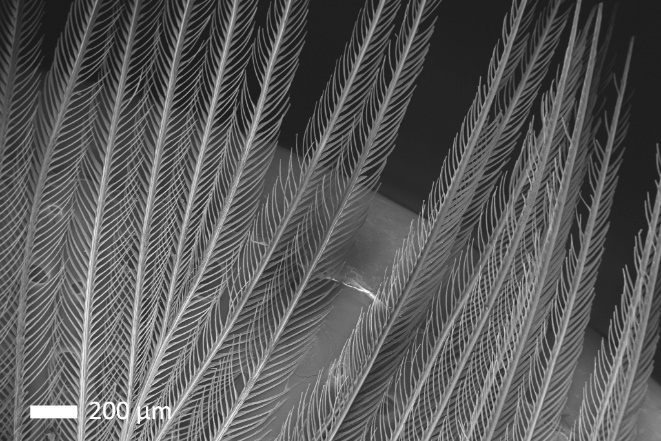

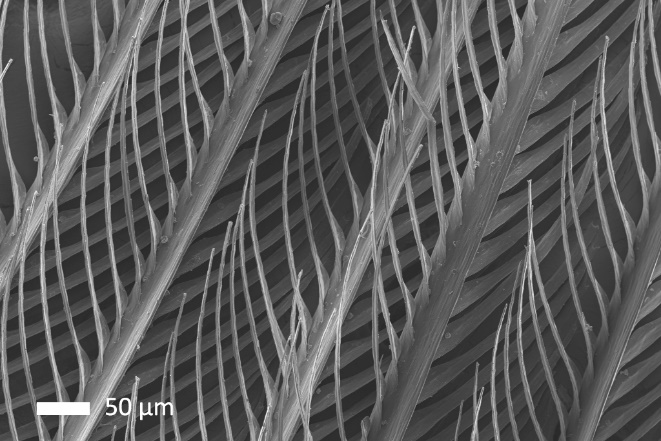


Male rump


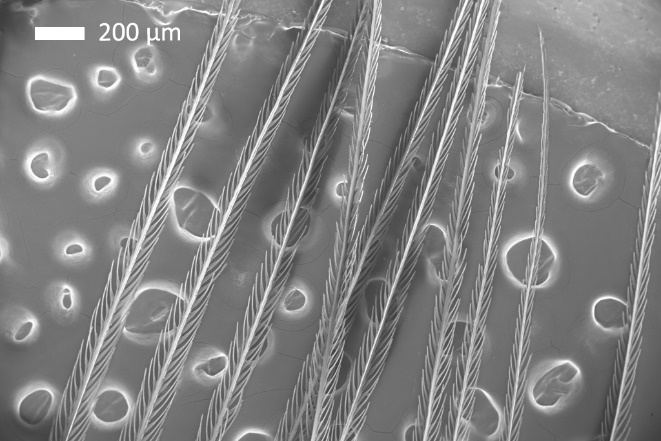

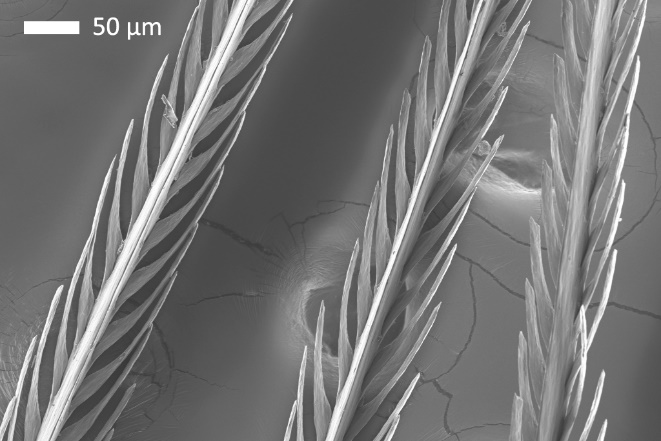


## B. Ramphocelus carbo

Female chest

*
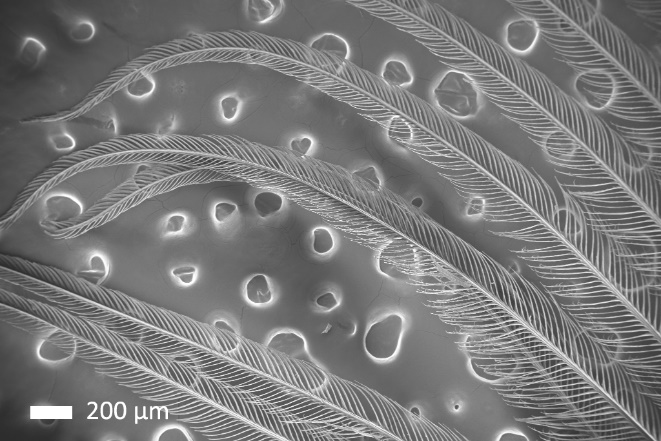

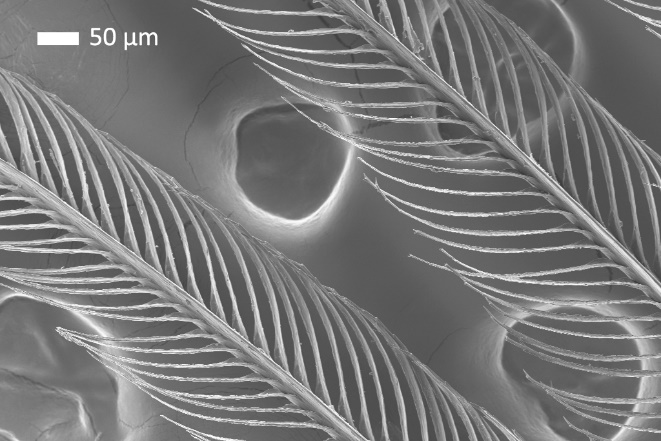
*

Male velvet red back:

*
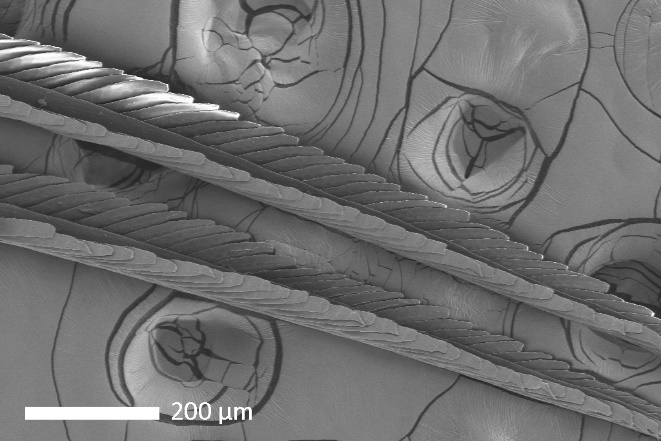

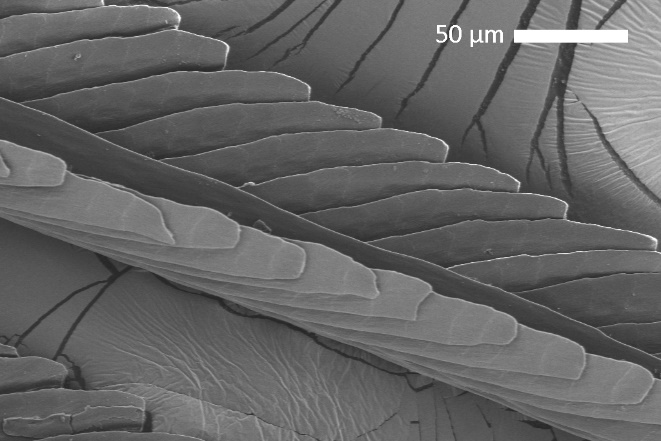
*

Red breast:

*
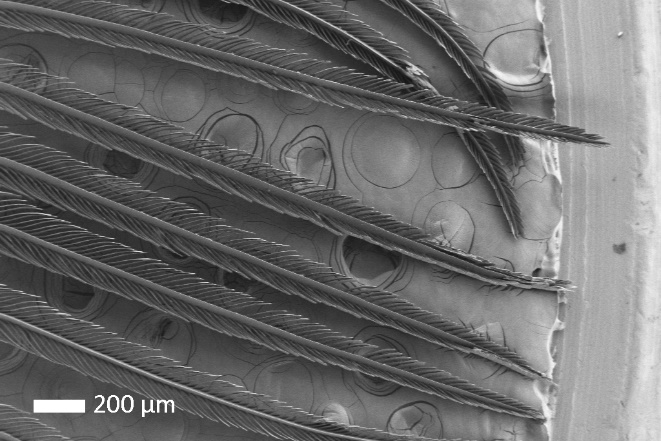

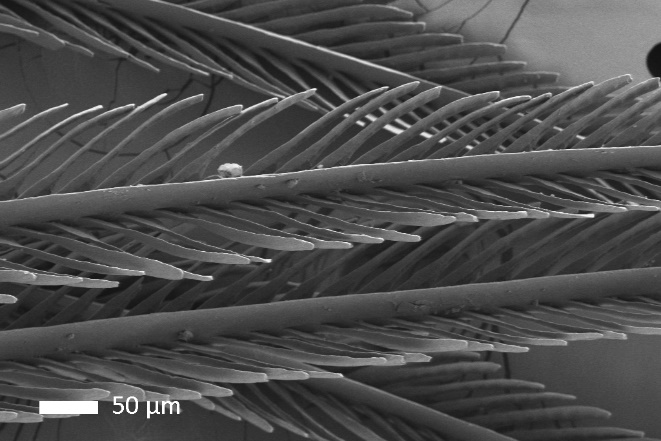
*

## C. Ramphocelus passerinii costaricensis

Female throat

*
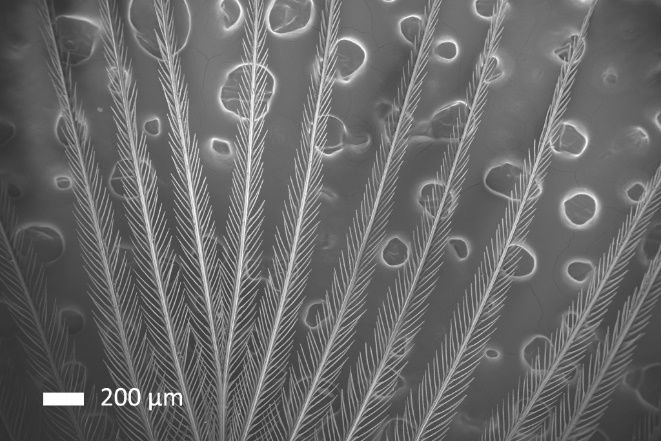

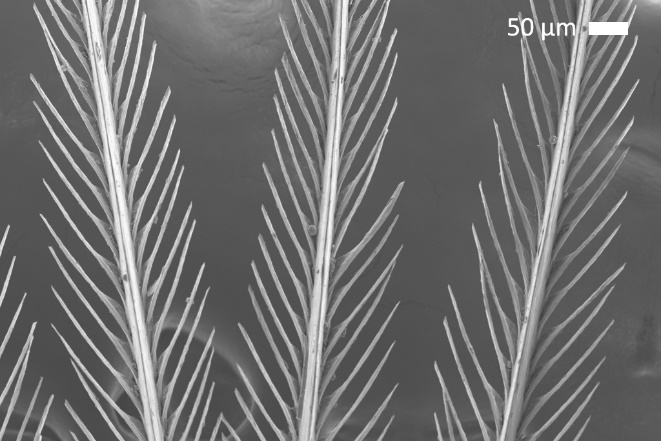
*

Male rump

*
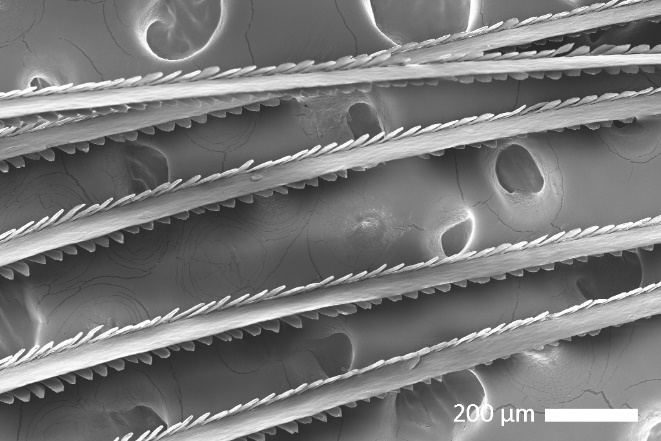

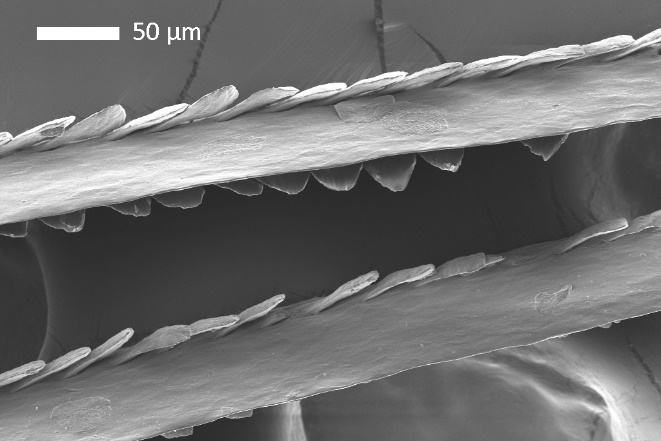
*

## D. Ramphocelus dimidiatus

Female rump:


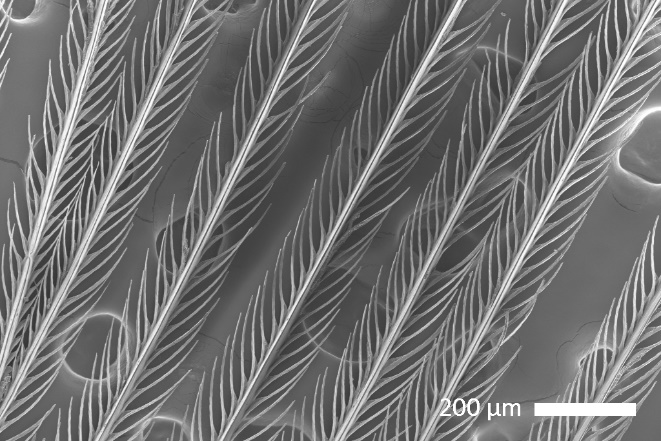

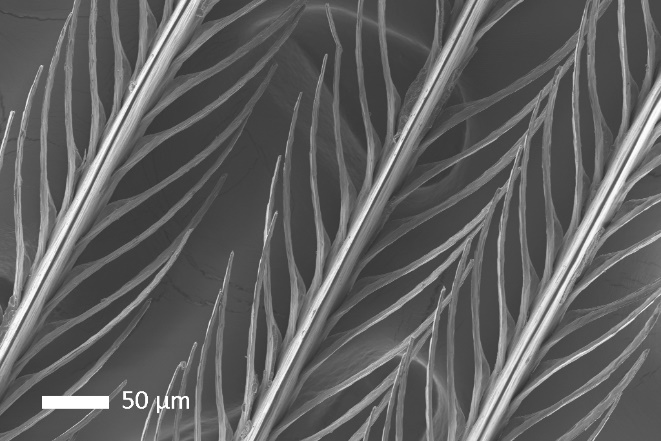


Female Throat:

*
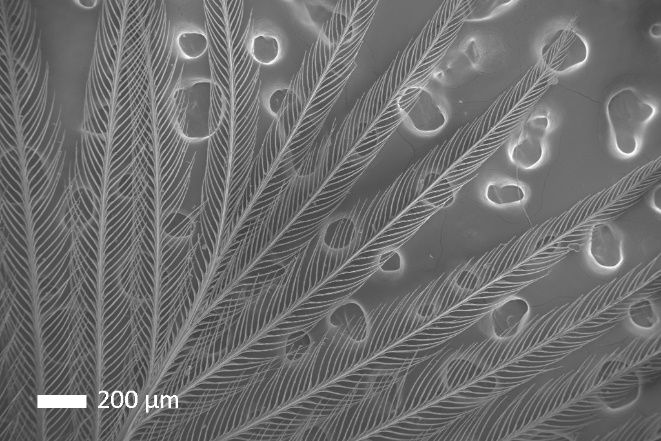

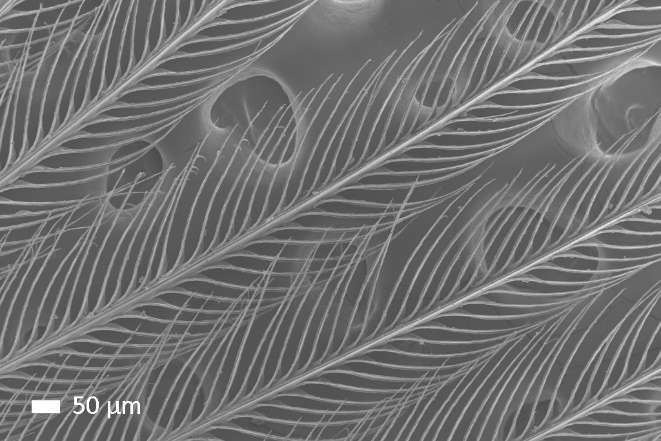
*

Male rump:

*
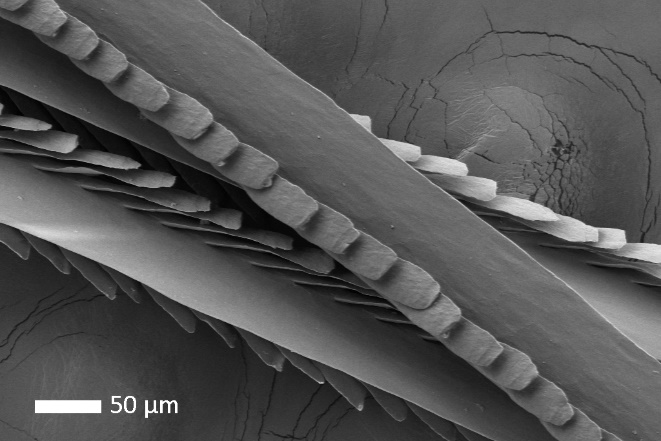

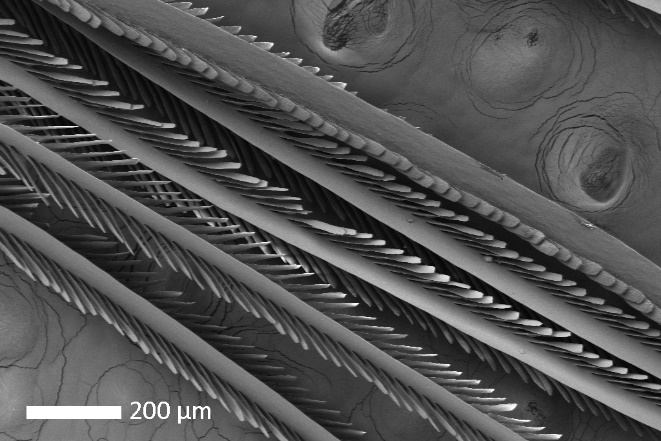
*

Male throat:

*
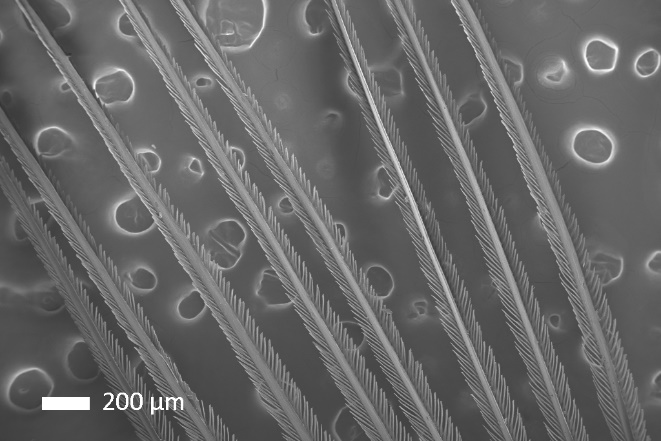

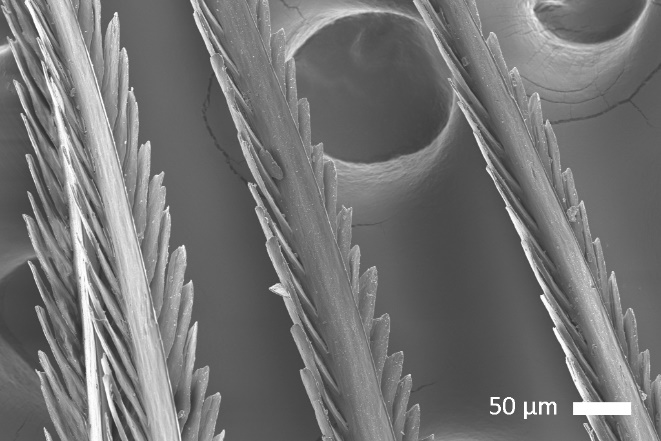
*

## E. Ramphocelus flammigerus

Female breast:


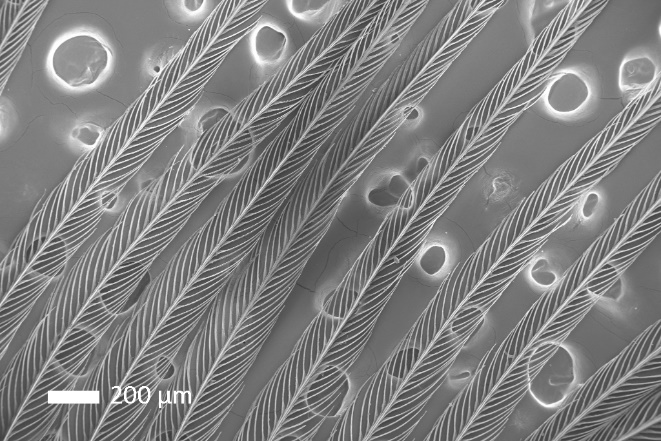

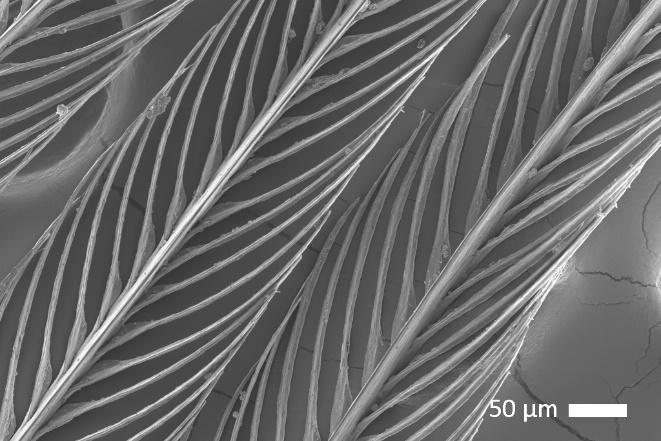


Female rump:

*
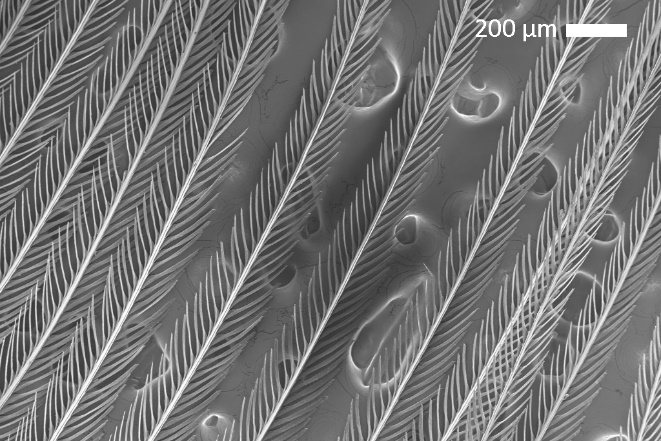

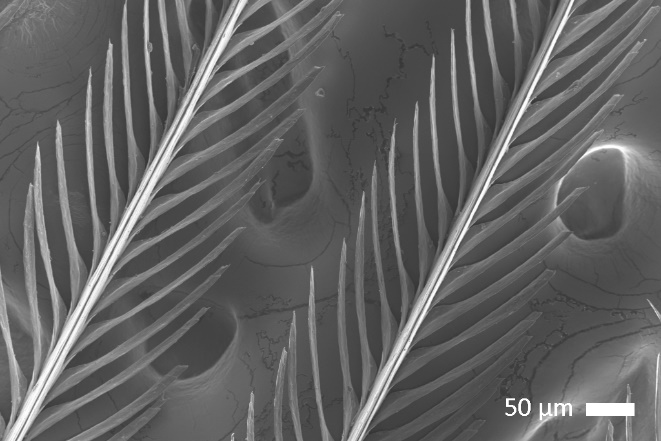
*

Male rump

*
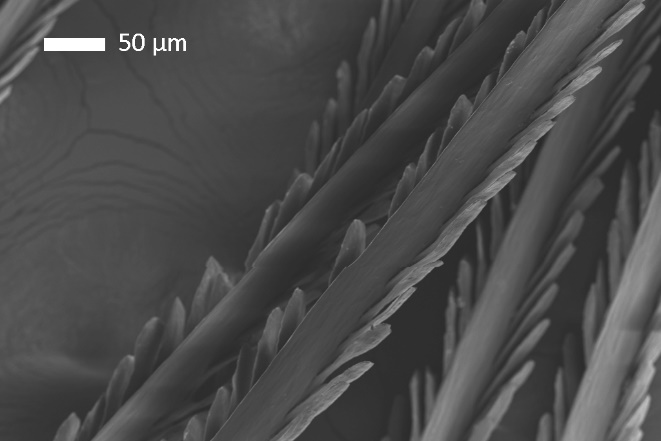

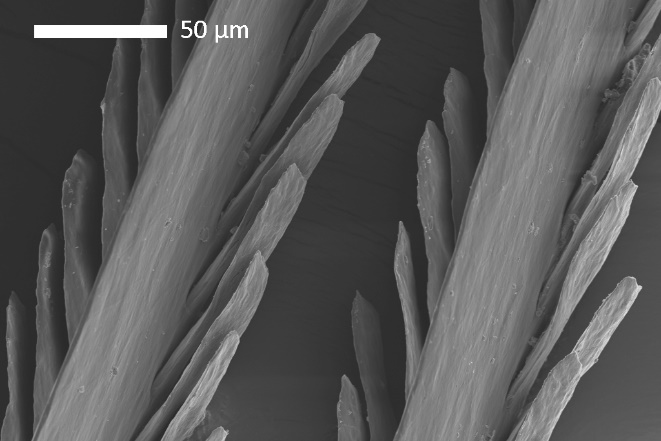
*

## F. Ramphocelus flammigerus icteronotus

Female rump

*
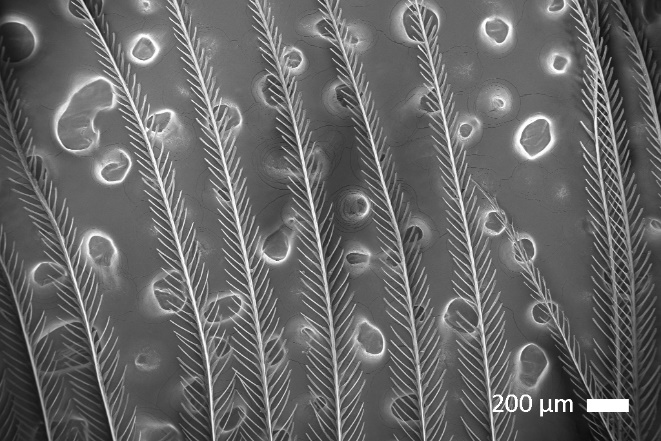

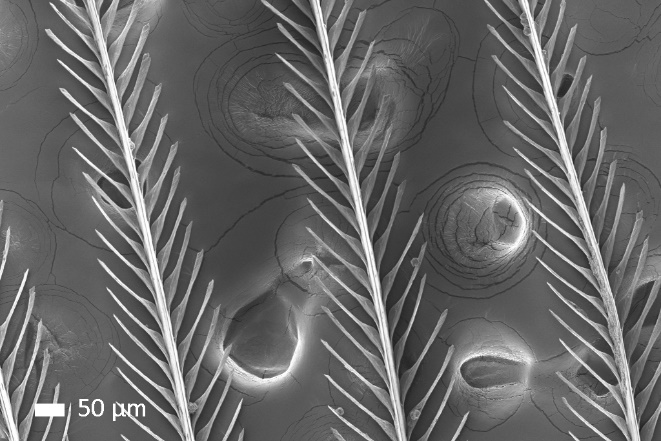
*

Male rump

*
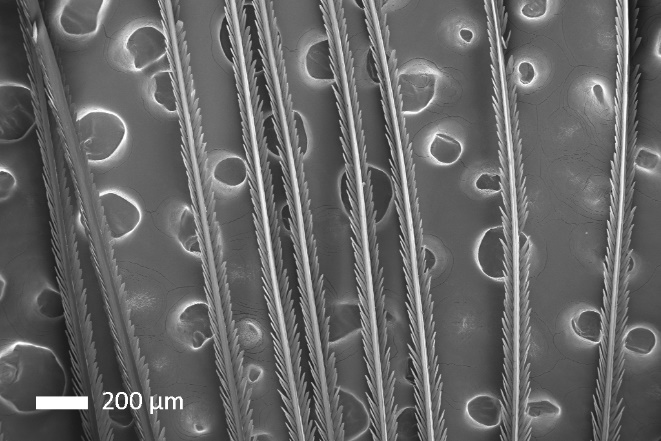

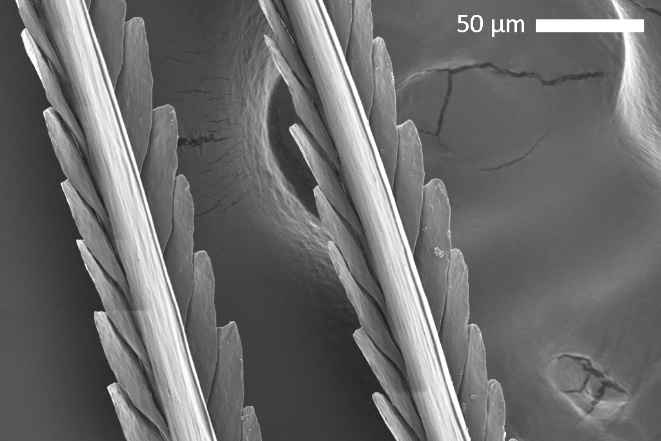
*

## G. Ramphocelus melanogaster

Female chest

*
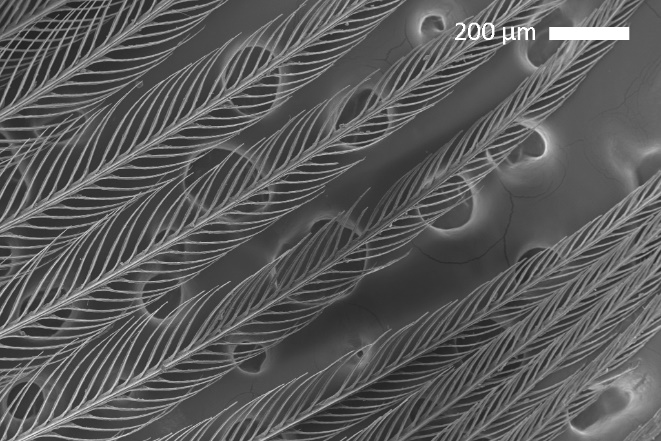

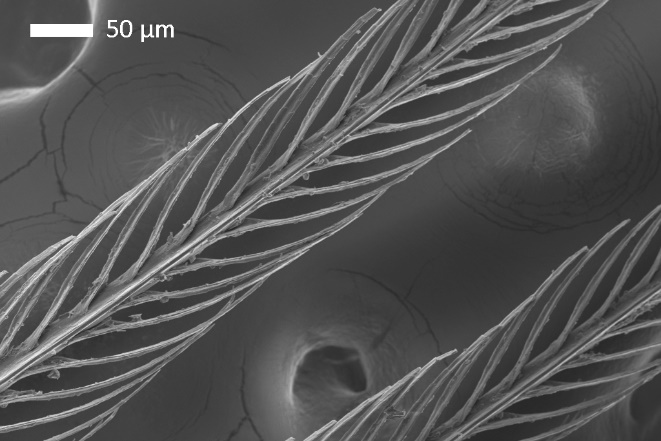
*

Male Throat Upper:

*
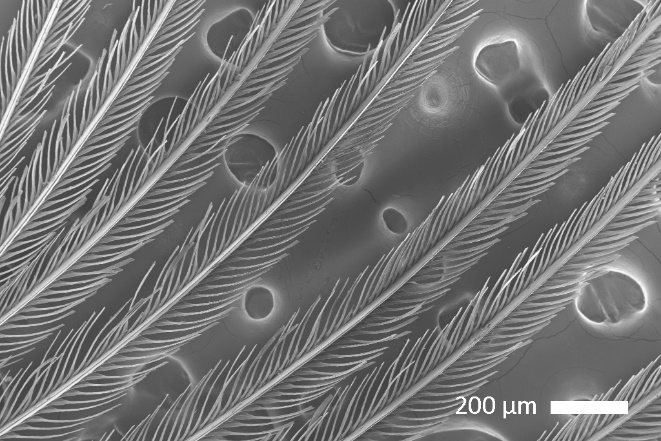

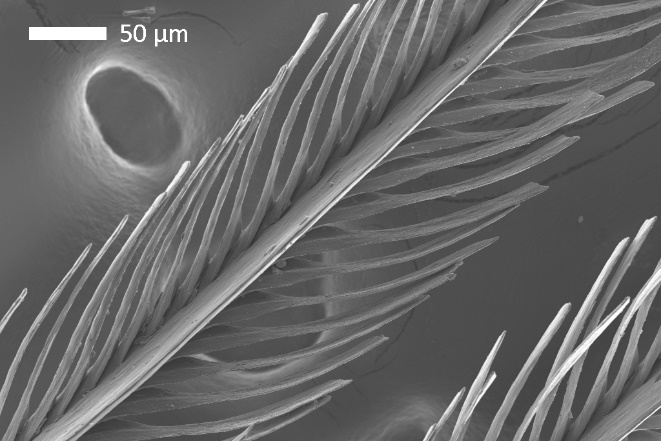
*

Male Throat Lower:

*
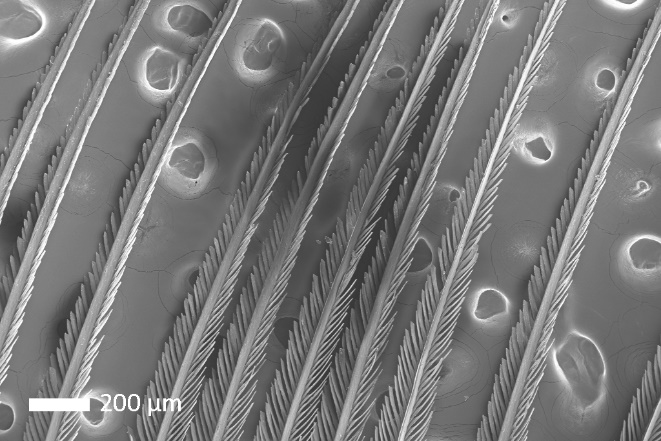

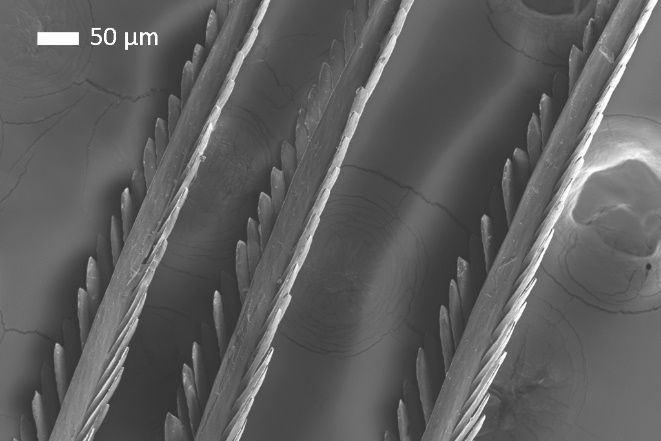
*

## H. Ramphocelus nigrogularis

Female rump

*
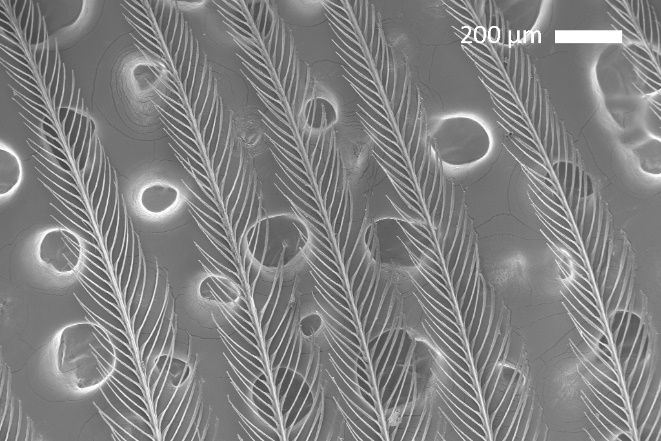

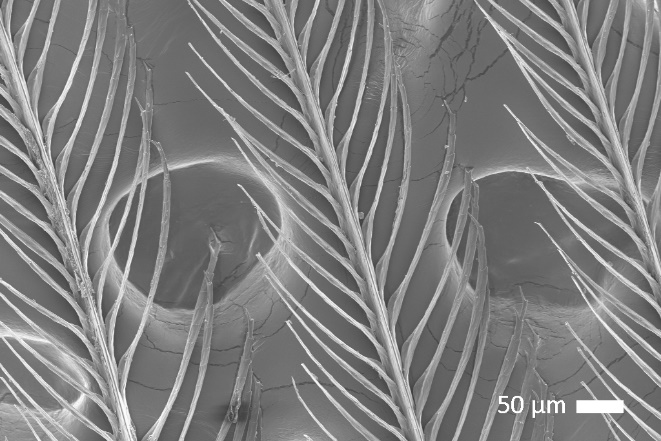
*

Male rump

*
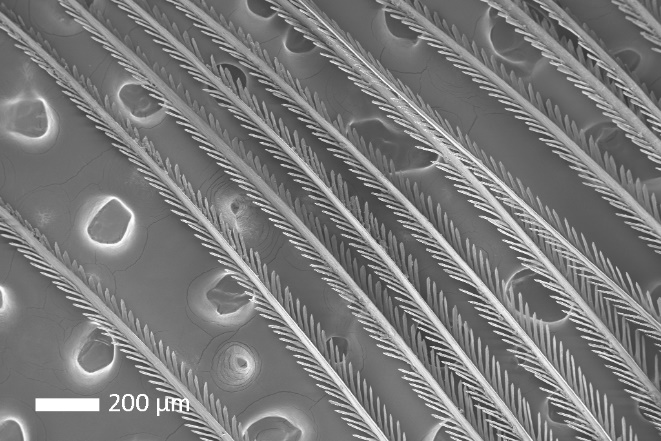

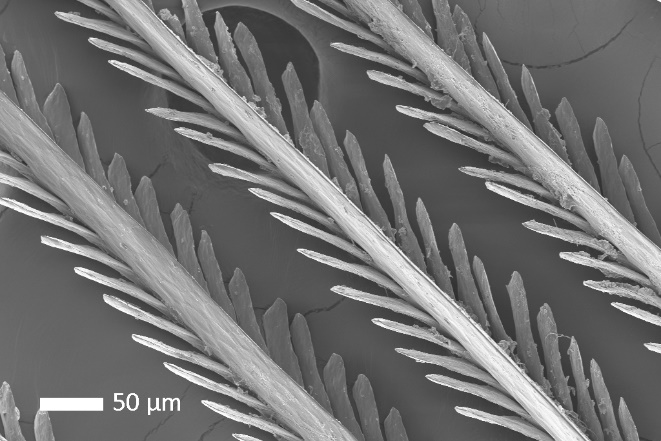
*

## I. Ramphocelus passerinii

Female rump

*
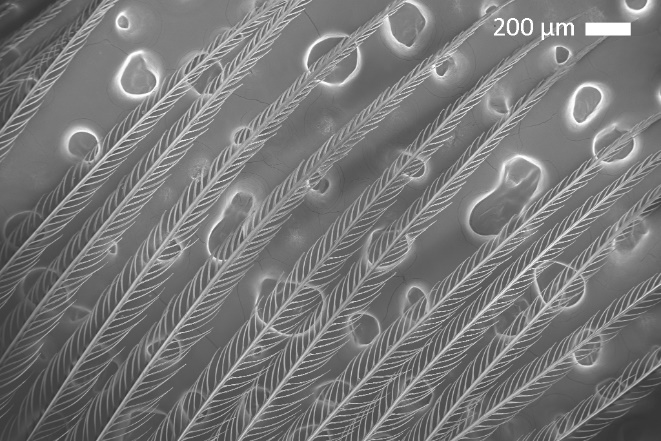

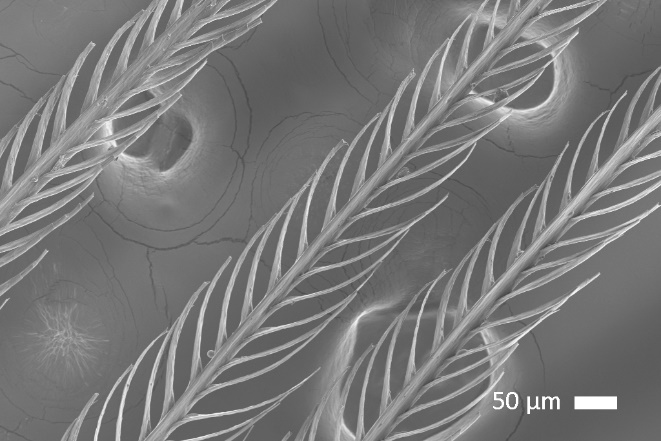
*

Male rump

*
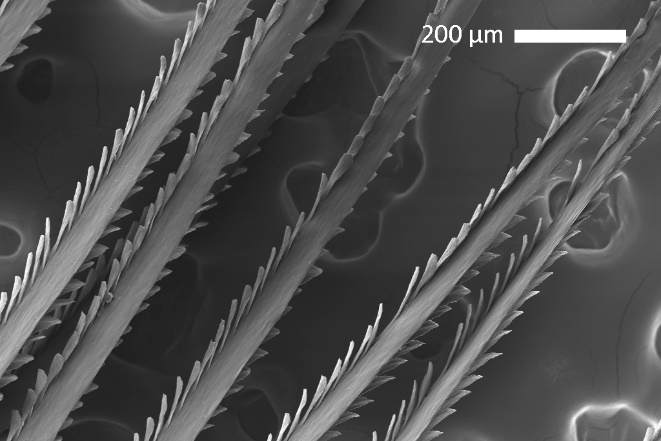

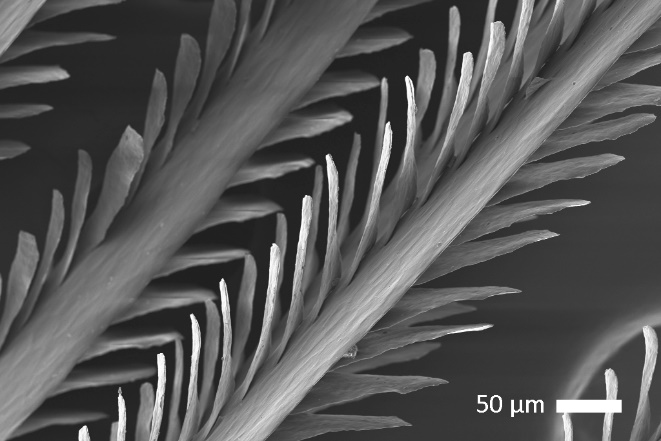
*

## J. Ramphocelus sanguinolentus

Female rump

*
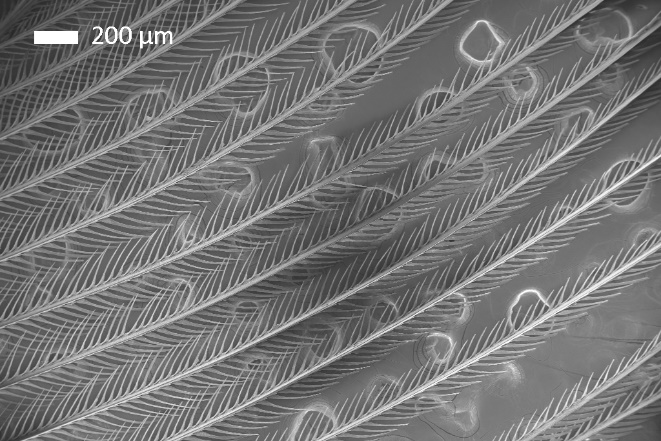

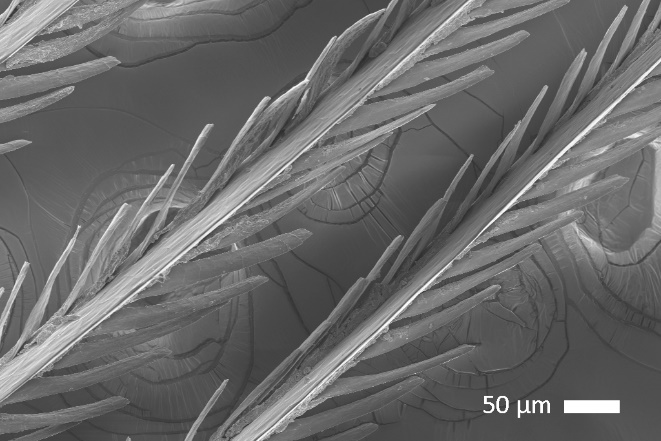
*

Male rump

*
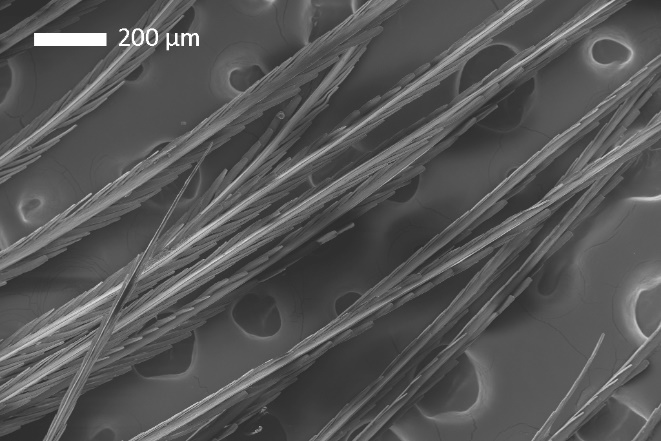

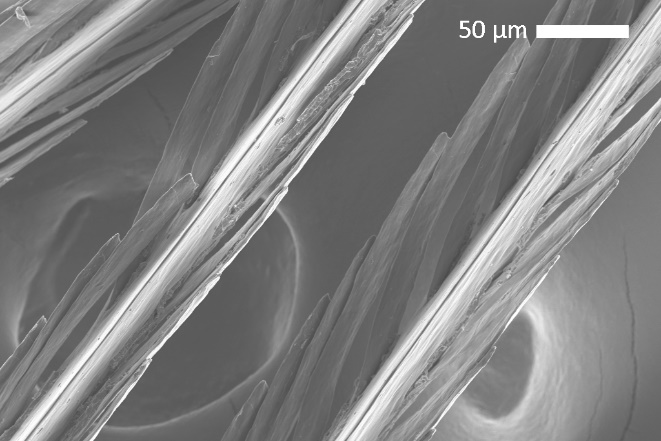
*

# Table S1: Specimen details.

| **scientific name** | **sex** | **specimen number** | **feather region** | **qualitative**  **patch color** |
| --- | --- | --- | --- | --- |
| *R. sanguinolentus* | M | MCZ 110057 | rump | red |
| *R.  sanguinolentus* | F | MCZ 110055 | rump | red |
| *R.  nigrogularis* | M | MCZ 139568 | rump | red |
| *R.  nigrogularis* | F | MCZ 299482 | rump | red |
| *R.  dimidiatus** | M | MCZ 105428, 105430, 105431 | throat | dark velvet red |
| *R.  dimidiatus** | M | MCZ 105428, 105430, 105431 | rump | bright red |
| *R.  dimidiatus* | F | MCZ 105445 | throat | dark red |
| *R.  dimidiatus* | F | MCZ 105445 | rump | red |
| *R.  melanogaster* | M | MCZ 96403 | throat (upper) | dark velvet red |
| *R.  melanogaster* | M | MCZ 96403 | throat (lower) | bright red |
| *R.  melanogaster* | F | MCZ 96404 | chest | dark red |
| *R.  melanogaster* | F | MCZ 96404 | throat | red |
| *R.  carbo** | M | MCZ 299748, 299750, 299751 | breast | dark red |
| *R. carbo* | M | MCZ 299751 | back | dark red-black velvet |
| *R.  carbo* | F | MCZ 299749 | chest | dark red |
| *R.  bresilius* | M | MCZ 273881 | rump | red |
| *R.  bresilius* | F | MCZ 273880 | rump | red |
| *R. costaricensis* | M | MCZ 108121 | rump | red-orange |
| *R. costaricensis* | F | MCZ 108141 | throat | orange-yellow |
| *R.   costaricensis* | F | MCZ 108141 | rump | orange |
| *R.  passerinii* | M | MCZ 110096 | rump | red |
| *R.  passerinii* | F | MCZ 110103 | rump | reddish-orange |
| *R.  flammigerus* | M | MCZ 103856 | rump | orange |
| *R.  flammigerus* | F | MCZ 103892 | breast | reddish-orange |
| *R.  flammigerus* | F | MCZ 103892 | rump | orange-yellow |
| *R.  flammigerus icteronotus* | M | MCZ 107435 | rump | yellow |
| *R.  flammigerus icteronotus* | F | MCZ 107448 | rump | yellow |

**Table S1:** **Specimen Details.** Specimens used in pigment extractions and SEM imaging. * indicates species for whom multiple individuals were combined for pigment extraction (to determine minimum amount of feather necessary). MCZ refers to the Harvard Museum of Comparative Zoology.

# Table S2: NCBI Accession Numbers.

| **species** | **NCBI accession number** |
| --- | --- |
| *Ramphocelus carbo* | U15723 |
| *Ramphocelus melanogaster* | FJ799883 |
| *Ramphocelus bresilius* | U15724 |
| *Ramphocelus dimidiatus* | FJ799881 |
| *Ramphocelus nigrogularis* | U15721 |
| *Ramphocelus costaricensis* | U15722 |
| *Ramphocelus passerinii* | EF529965 |
| *Ramphocelus flammigerus* | KR817429 |
| *Ramphocelus f. icteronotus* | U15719 |
| *Ramphocelus sanguinolentus* | U15718 |
| *Tachyphonus coronatus* | FJ799885 |
| *Tachyphonus rufus* | FJ799896 |
| *Tachyphonus phoenicius* | FJ799893 |
| *Eucometis penicillata* | FJ799875 |
| *Lanio fulvus* | EU647917 |
| *Tachyphonus luctuosus* | EF529967 |

# Table S3: Microstructural measurements

| **species** | **sex** | **region** | **barb width (maximum)** | | **barb width (top-down)** | | **barbule width** | | **inter-barbule distance (for 5 barbules)** | | **barb-barbule angle** | | **barbule length** | |
| --- | --- | --- | --- | --- | --- | --- | --- | --- | --- | --- | --- | --- | --- | --- |
|  |  |  | **mean** | **sd** | **mean** | **sd** | **mean** | **sd** | **mean** | **sd** | **mean** | **sd** | **mean** | **sd** |
| *R. bresilius* | f | rump | 30.78 | 2.71 | 26.30 | 1.42 | 5.35 | 0.78 | 195.89 | 11.16 | 31.10 | 3.36 | 227.29 | 15.39 |
| *R. carbo* | f | chest | 21.01 | 1.83 | 16.33 | 2.14 | 6.63 | 1.36 | 175.67 | 12.02 | 39.95 | 3.85 | 232.95 | 23.18 |
| *R. costaricensis* | f | throat | 21.96 | 2.69 | 20.45 | 1.16 | 5.88 | 1.41 | 200.95 | 12.98 | 27.52 | 2.94 | 167.01 | 6.55 |
| *R. dimidatus* | f | rump | 22.76 | 2.30 | 20.61 | 1.36 | 5.83 | 1.10 | 213.06 | 23.63 | 28.99 | 4.71 | 161.36 | 13.38 |
| *R. dimidatus* | f | throat | 20.00 | 2.97 | 16.55 | 2.76 | 6.50 | 1.98 | 227.87 | 15.98 | 35.87 | 3.64 | 284.49 | 25.91 |
| *R. flammigerus* | f | breast | 18.17 | 1.45 | 16.74 | 2.29 | 6.77 | 2.13 | 230.08 | 26.58 | 29.05 | 3.99 | 227.35 | 11.58 |
| *R. flammigerus* | f | rump | 18.22 | 1.14 | 18.14 | 2.89 | 7.70 | 2.22 | 236.94 | 12.77 | 31.37 | 4.86 | 191.65 | 6.88 |
| *R. f. icteronotus* | f | rump | 18.22 | 0.86 | 16.19 | 1.90 | 7.27 | 2.33 | 263.52 | 7.25 | 35.79 | 5.92 | 146.51 | 18.61 |
| *R. melanogaster* | f | chest | 19.76 | 1.30 | 17.08 | 2.07 | 5.94 | 1.43 | 243.24 | 9.65 | 22.43 | 1.09 | 171.70 | 12.09 |
| *R. melanogaster* | f | throat | 22.31 | 2.49 | 20.67 | 2.08 | 6.50 | 1.14 | 189.69 | 5.93 | 32.66 | 1.73 | 196.60 | 27.31 |
| *R. nigrogularis* | f | rump | 19.89 | 1.62 | 15.51 | 0.96 | 5.78 | 2.24 | 208.80 | 16.61 | 28.14 | 2.86 | 232.46 | 25.80 |
| *R. passerinii* | f | rump | 23.17 | 4.17 | 17.96 | 1.28 | 6.80 | 1.47 | 230.51 | 6.59 | 30.15 | 2.52 | 157.49 | 22.09 |
| *R. sanguinolentis* | f | rump | 31.70 | 4.47 | 23.22 | 3.72 | 12.14 | 2.39 | 249.25 | 20.78 | 18.40 | 3.91 | 136.49 | 22.73 |
| *R. bresilius* | m | rump | 22.29 | 1.83 | 15.25 | 1.21 | 9.97 | 1.31 | 258.94 | 17.94 | 22.66 | 5.87 | 121.23 | 13.79 |
| *R. carbo* | m | back | 42.89 | 4.15 | 14.85 | 1.77 | 17.45 | 0.79 | 169.31 | 10.59 | 24.97 | 11.17 | 162.93 | 11.65 |
| *R. carbo* | m | breast | 36.59 | 1.91 | 19.76 | 2.50 | 13.40 | 2.41 | 179.82 | 11.91 | 29.08 | 9.00 | 124.68 | 17.29 |
| *R. costaricensis* | m | rump | 43.45 | 1.04 | 42.26 | 3.40 | 24.80 | 3.15 | 176.38 | 12.49 | 15.16 | 4.29 | 60.31 | 4.94 |
| *R. dimidatus* | m | throat | 32.98 | 3.00 | 32.37 | 10.63 | 13.79 | 3.23 | 163.03 | 11.11 | 14.38 | 1.66 | 78.85 | 14.92 |
| *R. dimidiatus* | m | rump | 81.43 | 6.27 | 45.36 | 5.30 | 13.41 | 1.85 | 213.76 | 5.14 | 20.48 | 3.94 | 101.00 | 13.24 |
| *R. flammigerus* | m | rump | 36.00 | 1.62 | 32.50 | 0.85 | 11.91 | 2.02 | 164.69 | 6.36 | 19.28 | 6.66 | 87.66 | 9.67 |
| *R. f. icteronotus* | m | rump | 27.85 | 2.05 | 14.00 | 9.81 | 16.24 | 3.21 | 174.24 | 8.09 | 15.88 | 5.42 | 63.79 | 2.98 |
| *R. melanogaster* | m | throat | 43.69 | 3.11 | 21.25 | 1.55 | 7.82 | 1.77 | 158.56 | 11.94 | 30.03 | 6.96 | 175.78 | 8.49 |
| *R. melanogaster* | m | throat | 39.35 | 0.98 | 27.92 | 1.35 | 15.35 | 1.29 | 172.80 | 19.30 | 22.81 | 2.74 | 85.85 | 16.76 |
| *R. nigrogularis* | m | rump | 29.45 | 1.06 | 28.45 | 2.17 | 12.93 | 3.80 | 185.56 | 11.12 | 25.08 | 2.17 | 87.65 | 5.79 |
| *R. passerinii* | m | rump | 49.22 | 6.93 | 43.67 | 6.87 | 19.06 | 1.57 | 205.95 | 8.88 | 26.82 | 4.23 | 110.98 | 9.52 |
| *R. sanguinolentis* | m | rump | 32.84 | 1.72 | 22.18 | 2.46 | 18.98 | 0.61 | 327.04 | 19.86 | 19.07 | 3.51 | 196.91 | 11.82 |

**Table S3: Microstructural measurements.** Measurements of SEM photos for all feathers from all species in µm.

# Table S4: Pigment identification using LC-MS.

| **pigment family** | **molecule name** | **monoisotopic mass** | **formula** | **retention time (min)** | **identification** |
| --- | --- | --- | --- | --- | --- |
| apo-8-carotenal | apo-8-carotenal | 416.32 | C_30_H_40_O | 4.8 | matched pigment standard |
| β-Cryptoxanthin | β -Cryptoxanthin_1 | 552.43 | C_40_H_56_O | 3.8 | inferred |
|  | β -Cryptoxanthin_2 | 552.43 | C_40_H_56_O | 4.1 | inferred |
|  | β -Cryptoxanthin_3 | 552.43 | C_40_H_56_O | 4.4 | inferred |
|  | β -Cryptoxanthin_4 | 552.43 | C_40_H_56_O | 4.8 | inferred |
|  | β -Cryptoxanthin_5 | 552.43 | C_40_H_56_O | 5.7 | inferred |
| canthaxanthin | canthaxanthin_1 | 564.40 | C_40_H_52_O_2_ | 3.15 | possible isomer |
|  | canthaxanthin_2 | 564.40 | C_40_H_52_O_2_ | 3.4 | possible isomer |
|  | canthaxanthin_3 | 564.40 | C_40_H_52_O_2_ | 3.7 | possible isomer |
|  | canthaxanthin | 564.40 | C_40_H_52_O_2_ | 4.3 | matched pigment standard |
|  | canthaxanthin.isomer | 564.40 | C_40_H_52_O_2_ | 4.8 | isomer of pigment standard |
| zeaxanthin | zeaxanthin_1 | 568.43 | C_40_H_56_O_2_ | 3.6 | inferred |
|  | zeaxanthin_2 | 568.43 | C_40_H_56_O_2_ | 3.8 | inferred |
|  | zeaxanthin_3 | 568.43 | C_40_H_56_O_2_ | 4.6 | inferred |
|  | zeaxanthin_4 | 568.43 | C_40_H_56_O_2_ | 5 | inferred |
|  | zeaxanthin_5 | 568.43 | C_40_H_56_O_2_ | 5.3 | inferred |
|  | zeaxanthin_6 | 568.43 | C_40_H_56_O_2_ | 6.3 | inferred |
| adonirubin | adonirubin_1 | 580.40 | C_40_H_52_O_3_ | 2.9 | inferred |
|  | adonirubin_2 | 580.40 | C_40_H_52_O_3_ | 3.4 | inferred |
|  | adonirubin_3 | 580.40 | C_40_H_52_O_3_ | 3.8 | inferred |
|  | adonirubin_4 | 580.40 | C_40_H_52_O_3_ | 4.6 | inferred |
| adonixanthin | adonixanthin_1 | 582.41 | C_40_H_54_O_3_ | 3.2 | inferred |
|  | adonixanthin_2 | 582.41 | C_40_H_54_O_3_ | 3.4 | inferred |
|  | adonixanthin_3 | 582.41 | C_40_H_54_O_3_ | 3.7 | inferred |
|  | adonixanthin_4 | 582.41 | C_40_H_54_O_3_ | 4.31 | inferred |
| didehydroastaxanthin | didehydroastaxanthin |  | C_40_H_50_O_4_ | 2.5 | presumed |
| Astaxanthin | astaxanthin | 596.39 | C_40_H_52_O_4_ | 2.8 | matched pigment standard |
|  | astaxanthin_1 | 596.39 | C_40_H_52_O_4_ | 3.1 | possible isomer |
|  | astaxanthin.isomer | 596.39 | C_40_H_52_O_4_ | 3.3 | isomer of pigment standard |

**Table S4: Pigment identification using LC-MS.** All of the carotenoid molecules found in *Ramphocelus* tanagers were identified in one of three ways (see “identification” column). “Matched pigment standard” indicates that the molecule matched a pigment standard run simultaneously with the samples. “Isomer of pigment standard” indicates that the molecule was identifiable as an isomer of that pigment standard. “Possible isomer” indicates that it is likely to be an isomer of the pigment standard based on similar retention time and MS/MS spectra. “Inferred” indicates a standard was not available for the family, and the identity was inferred based on accurate mass, retention time, MS/MS spectra comparison with libraries, and pigments commonly found in bird feathers as described in the literature.

# Table S5: PCA Loadings for microstructure PCAs (normal and phylogenetic).

|  | **normal PCA (all)** | | **phyloPCA (males)** | | **phyloPCA (females)** | |
| --- | --- | --- | --- | --- | --- | --- |
|  | **PC1** | **PC2** | **PC1** | **PC2** | **PC1** | **PC2** |
| **barb width (maximum)** | -0.31 | 0.53 | 0.881427 | 0.44595 | -0.63566 | 0.727779 |
| **barb width (top-down)** | -0.39 | -0.10 | 0.837797 | -0.45126 | -0.50981 | 0.7588 |
| **barbule width** | -0.42 | 0.06 | 0.299406 | -0.14884 | -0.83465 | -0.36525 |
| **inter-barbule distance** | 0.19 | -0.24 | -0.19279 | 0.591586 | -0.57043 | -0.63815 |
| **barb-barbule angle** | 0.39 | 0.26 | 0.013186 | 0.602456 | 0.873479 | -0.19204 |
| **barbule length** | 0.43 | 0.15 | -0.2042 | 0.950605 | 0.725528 | 0.418 |
| **barb oblongness (top-down/maximum)** | 0.01 | 0.74 |  |  |  |  |
| **barbule width over length** | -0.46 | -0.05 |  |  |  |  |

# Table S6: Optical power transmission results for simulations of oblong expanded barbs.

A one-way paired t-test (alternative = “less”) found that male-type feathers had significantly more optical power transmitted through the feather than female-type feathers (p = 0.0021, mean of difference = -125.9, 95% CI = [-Inf, -64.2]).

| **simulation** | **barb region** | **optical power transmission (W/m^2^)** | | **% difference** |
| --- | --- | --- | --- | --- |
|  |  | **female** | **male** |  |
| **truncated feather** | 45 | 210.2 | 266.4 | 26.8 % |
|  | center | 447.6 | 788.2 | 76.1 % |
|  | side | 92 | 134.7 | 46.4 % |
|  | top | 259.4 | 403.2 | 55.4 % |
| **full feather** | 45 | 210.9 | 266.7 | 26.4 % |
|  | center | 452.4 | 788.1 | 74.2 % |
|  | side | 92.8 | 135.9 | 46.4 % |
|  | top | 265.5 | 403.2 | 51.9 % |
| **vacuole** | 45 | 210.8 | 267.8 | 27 % |
|  | side | 219.7 | 254.5 | 15.8 % |
|  | top | 262.9 | 399.9 | 52.1 % |
